# Comparative genome analysis of the vineyard weed endophyte *Pseudomonas viridiflava* CDRTc14 showing selective herbicidal activity

**DOI:** 10.1038/s41598-017-16495-y

**Published:** 2017-12-11

**Authors:** Abdul Samad, Livio Antonielli, Angela Sessitsch, Stéphane Compant, Friederike Trognitz

**Affiliations:** AIT Austrian Institute of Technology, Center for Health and Bioresources, Bioresources Unit, Konrad-Lorenz-Straße 24, A-3430 Tulln, Austria

## Abstract

Microbes produce a variety of secondary metabolites to be explored for herbicidal activities. We investigated an endophyte *Pseudomonas viridiflava* CDRTc14, which impacted growth of its host *Lepidium draba* L., to better understand the possible genetic determinants for herbicidal and host-interaction traits. Inoculation tests with a variety of target plants revealed that CDRTc14 shows plant-specific effects ranging from beneficial to negative. Its herbicidal effect appeared to be dose-dependent and resembled phenotypically the germination arrest factor of *Pseudomonas fluorescens* WH6. CDRTc14 shares 183 genes with the herbicidal strain WH6 but the formylaminooxyvinylglycine (FVG) biosynthetic genes responsible for germination arrest of WH6 was not detected. CDRTc14 showed phosphate solubilizing ability, indole acetic acid and siderophores production *in vitro* and harbors genes for these functions. Moreover, genes for quorum sensing, hydrogen cyanide and ACC deaminase production were also found in this strain. Although, CDRTc14 is related to plant pathogens, we neither found a complete pathogenicity island in the genome, nor pathogenicity symptoms on susceptible plant species upon CDRTc14 inoculation. Comparison with other related genomes showed several unique genes involved in abiotic stress tolerance in CDRTc14 like genes responsible for heavy metal and herbicide resistance indicating recent adaptation to plant protection measures applied in vineyards.

## Introduction

Worldwide, weeds compromise crop yield and increase costs of crop production^1^. The weed problem on global food production has become severe due to increased use of synthetic herbicides, the rapidly evolving weed resistance against herbicides and negative impacts on the environment^2,3^. The employment of bioherbicides within the framework of an integrated weed management strategy has the potential to offer a number of benefits such as higher target specificity, faster degradation and lower (or no) toxicity than chemical herbicides. Despite the efforts of many researchers to find new bioherbicidal candidates *in vitro* and to test them under field conditions, only thirteen authorized bioherbicides derived from microorganisms or natural molecules are currently available on the market. Among them, only three are based on bacteria, ten on fungi, one is based on a virus and one contains natural plant extracts^4,5^.

Bacterial herbicides have many benefits over fungal herbicides, particularly due to quick growth of bacteria, relatively simple propagation and storage conditions, and greater amenability to genetic manipulation^6,7^. Many bacterial species/strains have been investigated as natural weed control agents, especially *Pseudomonas fluorescens* and *Xanthomonas campestris*, but only *P*. *fluorescens* strain WH6 has been investigated to understand the genetic basis of its herbicidal activity^8^. A culture filtrate of strain WH6 arrests the germination of a number of weedy grass species, including the annual bluegrass (*Poa annua* L.)^9,10^. The herbicidal activity of strain WH6 has been attributed to the production of formylaminooxyvinylglycine (FVG), originally referred to as the germination arrest factor, and the *gvg* gene cluster^9,11^ was reported to be responsible for the production of FVG^11^.

*Pseudomonas viridiflava* is a pectinolytic bacterium in the *P*. *syringae* species complex. Strains belonging to this species generally show a broad host range and can live either as pathogen and saprophyte^12^. Pathogenic strains of *P*. *viridiflava* generally have two pathogenicity islands [single (S-PAI) and tripartite (T-PAI)] and/or the *avrE* effector of the type III secretion system^13^. Despite of being the disease causative agent of multiple plant species, some *P*. *viridiflava* strains exhibit biocontrol activities against various human and plant pathogenic fungi^14,15^. Additionally, some strains have been shown to degrade various pesticides (Fenvalerate, an organophosphate) and polycyclic aromatic hydrocarbons (PAHs)^16–18^. Although *P*. *viridiflava* belongs to the well-known *P*. *syringae* complex, the biology and functioning of this species is not thoroughly investigated and only nine draft *P*. *viridiflava* genomes are available in the NCBI database.

In this study we particularly addressed strain CDRTc14, which we previously isolated from the weed *Lepidium draba* L. and which showed inhibiting effects towards its host. Based on whole genome average nucleotide identity (ANI), CDRTc14 was assigned to *P*. *viridiflava* ^19^. As this strain is related to plant pathogens and inhibited growth of its plant host we aimed to better understand the activities of this strain, its interaction with plants and underlying mechanisms by employing in-depth genome analysis, comparative genomics and functional tests.

## Results and Discussion

### Herbicidal activity

**Figure 1:**
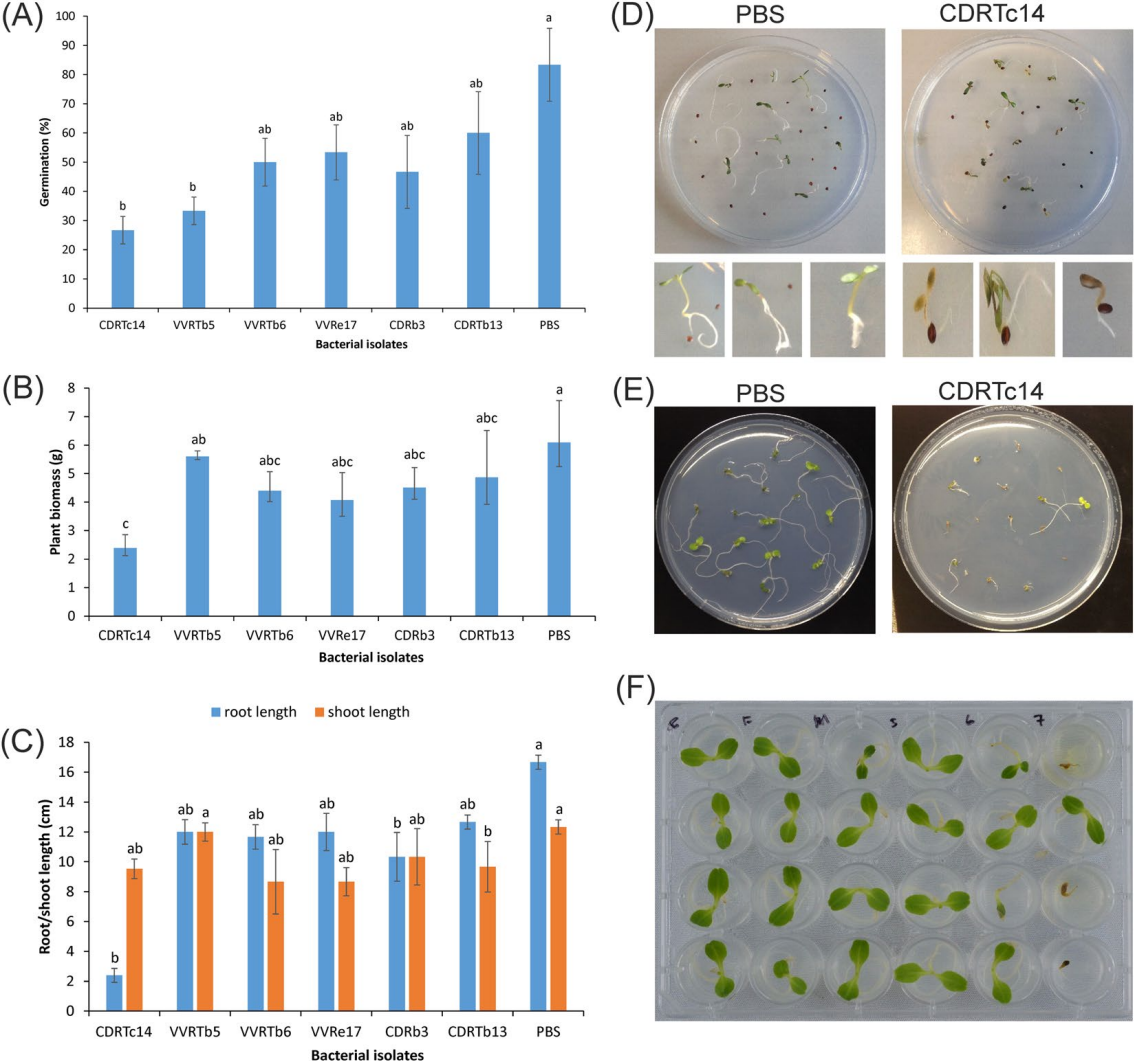
Bioherbicidal activity of CDRTc14 and other strains. (**A**–**C**) Results of greenhouse experiment showing germination percentage, plant biomass, root and shoot length. (**D**) Herbicidal effect of CDRTc14 on *L*. *draba* weed *in vitro* (**E**) Herbicidal effect of CDRTc14 on lettuce *in vitro*. (**F**) Dose dependent effect of CDRTc14 on lettuce, showing phytotoxic effect at 10^7^ CFU ml^−1^. PBS (Phosphate-buffered saline) was used as control.

Generally, bacterial herbicides are known to exhibit a narrow host range, and they impact mostly closely related plant species^8^. Here, we evaluated the herbicidal effect of bacteria isolated from *L*. *draba* on its host and closely related plant species. Overall 98 isolates were tested for their phytotoxic effect on *L*. *draba* but only six isolates (*P*. *viridiflava* CDRTc14, *P*. *fluorescens* CDRTb13, *Agromyces humatus* CDRb3, *Arthrobacter* sp. VVRe17, *Rhizobium* sp. VVRTb6, *Bosea* sp. VVRTb5, Table S6) showed growth inhibition on *L*. *draba in vitro* and under greenhouse conditions (Fig. 1). Among them, CDRTc14 showed the most pronounced effect on *L*. *draba* (both *in vitro* and greenhouse). Further investigation revealed that CDRTc14 has a negative effect on lettuce (*Lactuca sativa* L.) growth but not on *Arabidopsis thaliana* (Fig. S1). To obtain more information on the host range of CDRTc14, we tested its effect on nine different plant species, which are close relatives of *L*. *draba* and belong to the *Brassicaceae* family. Interestingly, we observed differential effects (including positive, neutral or negative) on these plant species due to CDRTc14 inoculation (Table 1). For example, CDRTc14 significantly inhibited (−56%) the germination of the weed *Sisymbrium officinale* (belonging to the *Brassicaceae* linage I (Fig. S3)) as compared to the mock treatment. However, this strain significantly enhanced root growth (69%) and plant biomass (52%) of the *Brassica napus* variety Cleopatra also belonging to the *Brassicaceae* linage I. In contrast, CDRTc14 significantly improved root length (76%) and seedling biomass (148%), respectively, of the phylogenetically closest relatives (linage III) *Lepidium meyenii* and *Erysimum odoratum*. CDRTc14 did not cause a significant effect on other *Brassicaceae* species (*B*. *napus* variety Pharao (linage I), *Hesperis matronalis* and *Lepidium sativum* (linage II). Our results are not in accordance with the centrifugal phylogenetic method (CPM)^20^, postulating that near-neighbor species to the target are at greater risk of attack than distant species. However, similar results were reported for the herbicidal strain of *Burkholderia andropogonis*, which showed differential effects on species of the *Caryophyllales* and *Fabaceae* family^21^. Also the biocontrol agent *P*. *fluorescens* strain D7 specifically inhibits downy brome growth while it stimulates the growth of rapeseed (*Brassica napus L*.)^22^. However, the CPM has been considered as the standard for host-specificity testing of biological control agents since 1974 but it has been suggested to revise this hypothesis^23^ and also our results contradict this hypothesis.

**Table 1:** Percent change in plant growth from different Brassicaceae with *P*. *viridiflava* CDRTc14 inoculation compared to uninoculated control.

Plant Species	Germination percentage	Root Length	Shoot Length	Seedling weight
*Lepidium meyenii*	−11.1	76.9**	0.7	44.0
*Hesperis matronalis*	−3.0	−19.2	−19.0	−34.5
*Isatis tinctoria*	−4.2	−26.7	5.9	1.0
*Erysimum odoratum*	35.7	−10.1	27.6	148**
*Sisymbrium officinale*	−56.3**	5.5	−1.3	−25.4
*Lepidium sativum*	8.3	17.1	5.2	3.2
*B. napus* *var*. Cleopatra	30.4	69.0**	−3.3	52.1*
*B*. *napus*. *var*. Pharao	−3.3	−2.8	−5.3	4.6

Note: Values in the table indicates percent change compare to untreated control. The Student’s T−tests was applied to compare treatment means with control (PBS) and significant values are indicated as *p < 0.05, **p < 0.01, ***p < 0.001.

### Genomic features and phylogenetic analysis

**Figure 2:**
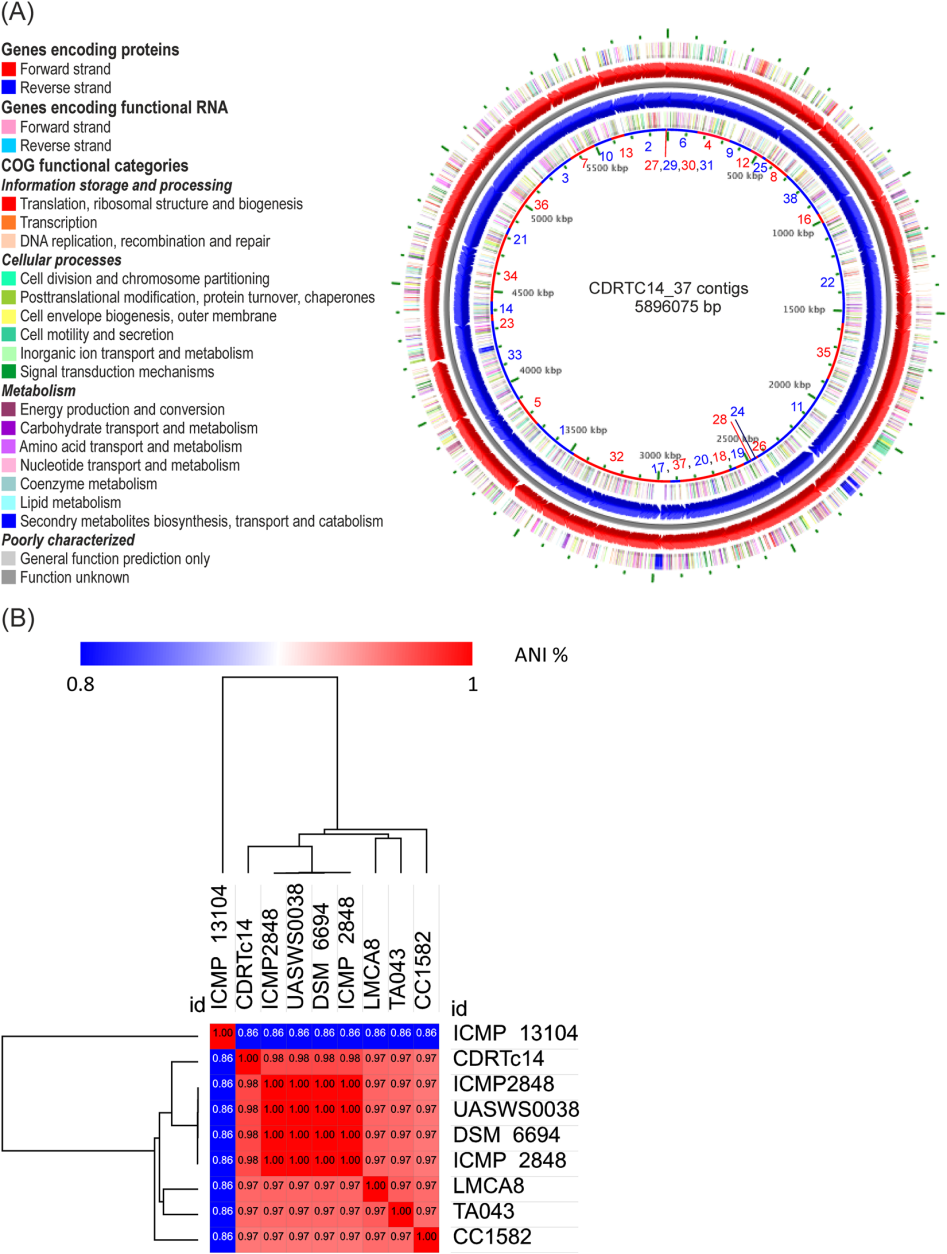
(**A**) Graphical circular map of *P*. *viridiflava* CDRTc14 with annotation. The innermost ring shows contig boundaries as alternating red and blue color where contig accession numbers MBPF01000001.1 to MBPF010000037.1 show the each contig position. (**B**) Heat-map of average nucleotide identity (ANI) values amongst different strains of *Pseudomonas viridiflava* revealing two major groups.

The CDRTc14 genome was sequenced using Illumina HiSeq and then assembled with SPAdes. The details about genome sequencing were reported earlier^19^ and genomic features are summarized in Tables S1 and S2. The whole genome of CDRTc14 has a total length of 5.96 Mb, a GC content of 59.3%, contains one chromosome and one plasmid (67,392 bp). The map of CDRTc14 genome with its annotation is represented in Fig. 2A. Genome relatedness of the strain CDRTc14 was analyzed on the basis of average nucleotide identity (ANI). Strains with ANI values >96% are considered to be the same species^24^. As shown in Fig. 2B, strain CDRTc14 has the greatest nucleotide identity (98.06%) with strain UASWS0038, which has been reported as a biocontrol agent against postharvest disease of pip fruits^25^. Other *P*. *viridiflava* genomes showed ANI values between 96.72% and 98.04% with CDRTc14 except ICMP_13104, which has only 85.90% ANI with CDRTc14. Low ANI similarity (less than 86%) of ICMP_13104 to all whole genome sequenced strains question its postulated taxonomic position as *P*. *viridiflava* ^26^.

### Identification of genes potentially involved in herbicide production

**Figure 3:**
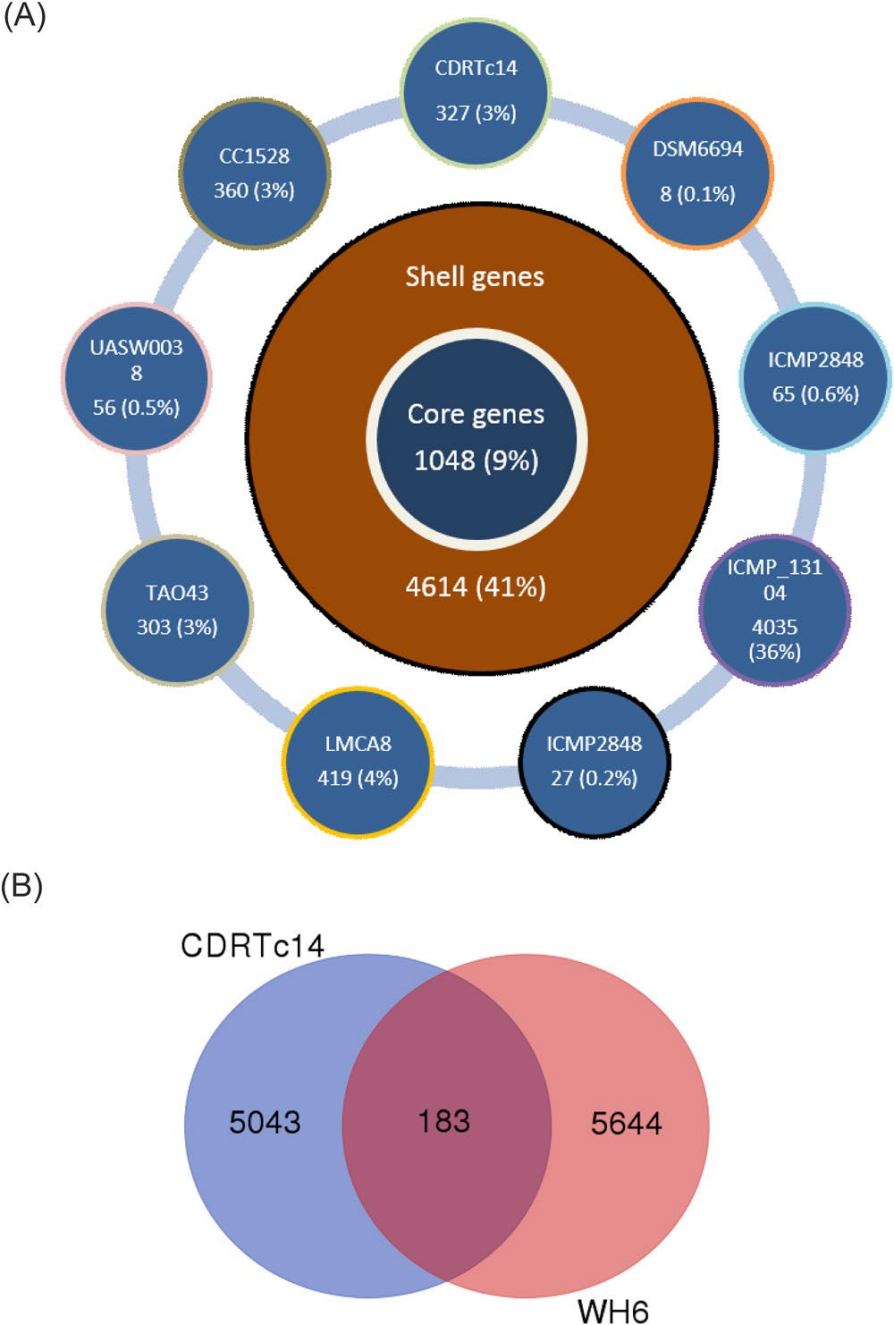
(**A**) Pan-genome of *P*. *viridiflava* strains calculated in Roary (with blastp at 95%). The inner ring shows the total number of the core genes (present in >99% of the genomes); the middle ring shows the number of shell genes (present in 15–95% of the genomes); the outer rings show the number of genes in the cloud of the pan-genome (present in 1–15% of the genomes), gene annotation of all genes is provided in Table S10. (**B**) Representing core and unique genes between *P*. *viridiflava* CDRTc14 and *P*. *fluorescens* WH6, annotation of core genes is provided in Table S9.

**Figure 4:**
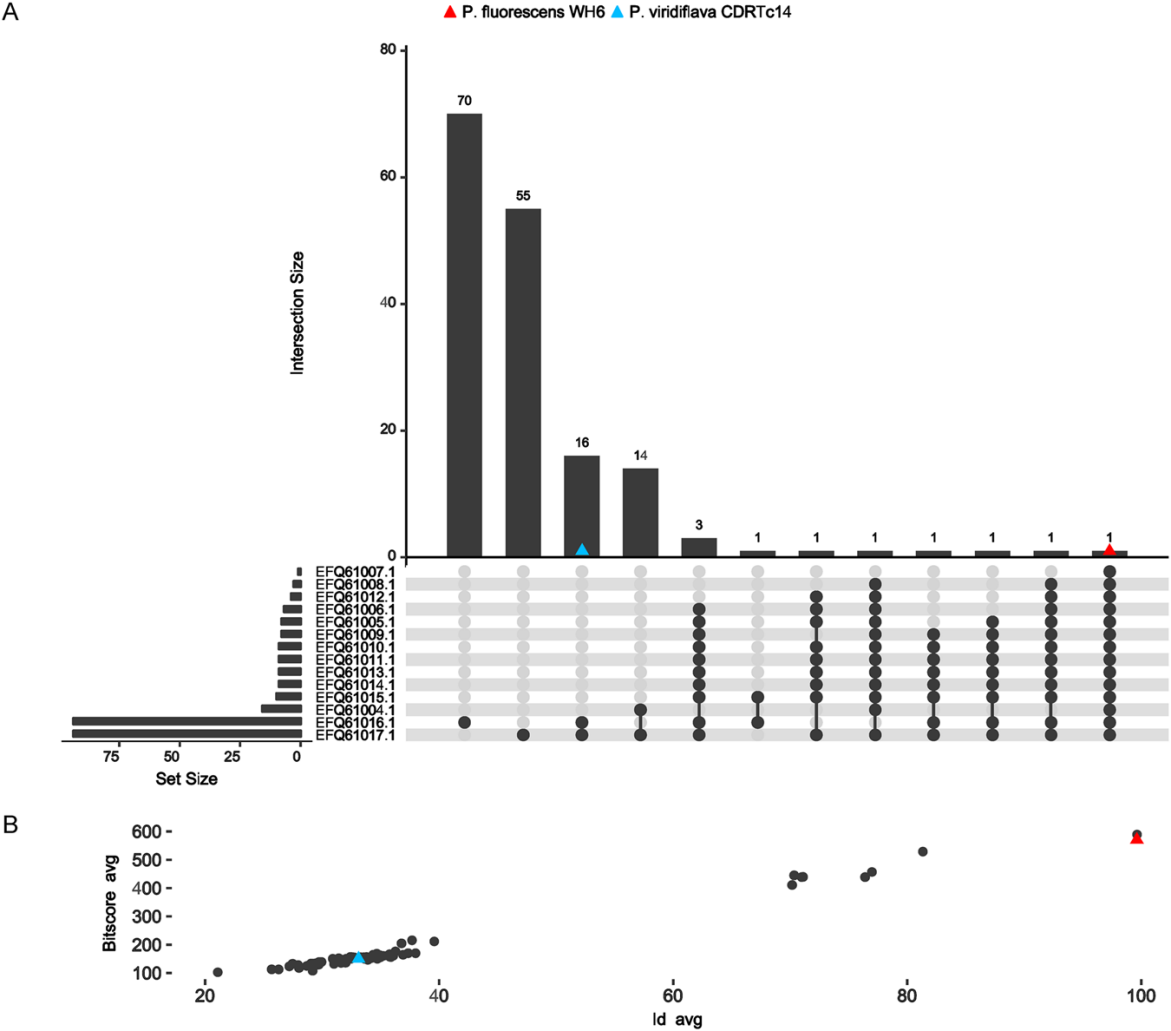
Occurrence of formylaminooxyvinylglycine (FVG) biosynthetic genes (*gvg* gene cluster of WH6) across tested genomes are shown by UpSet composite plot: (**A**) Intersection size vs set size: this plot shows the number of genomes (vertical barplots) sharing the same combination of *gvg* cluster elements (black dots read vertically) whereas the horizontal barplots report how many genomes have one specific element of the *gvg* cluster, gene by gene (black dots read horizontally). As expected, all the genes of the *gvg* cluster are present in *P*. *fluorescens* WH6 genome (red triangle). None of the other *Pseudomonas* genomes has the complete set of elements of the cluster (at least with an identity percentage ≥ 75%). *P*. *viridiflava* CDRTc14 shows only two genes and these are also the most abundant among *Pseudomonas genomes*. (**B**) Scatterplot showing the identity % average (x-axis) and the bitscore average (y-axis) for all the genes in the gvg cluster in each genome. P. fluorescens WH6 has an id avg of 100% and bitscore avg of 590, P. viridiflava CDRTc14 has an id avg of 32% and bitscore avg of 136.

Strain CDRTc14 showed similar phytotoxic effects on *L*. *draba* and lettuce seedlings *in vitro* as reported earlier for *P*. *fluorescens* strain WH6, which was correlated with the biosynthesis of 4-formylaminooxyvinylglycine (FVG) responsible for germination arrest of grasses^10^. The whole genomes of these two herbicidal strains (*P*. *viridiflava* CDRTc14 and *P*. *fluorescens* WH6) shared 183 genes (which correspond to 3.5% and 3.1% of all genes in CDRTc14 and WH6, respectively), while 5,043 and 5,644 genes were found specifically in CDRTc14 and WH6, respectively (Fig. 3B, Table S9). At the molecular level, the germination arrest factor of WH6 was reported to be encoded by the *gvg* cluster containing fourteen genes^9,11^. Eight genes (*gvgR*, *gvgA-C*, *gvgF-I*) of this cluster are involved in the FVG production and two genes (*gvgJ* and *gvgK*) are involved in the export of FVG while other genes (*gvgJ*, *gvgK*, *tam*, *ssb*) were shown not to be important for FVG production^11^. We investigated how commonly the *gvg* cluster (13–15 kbp) occurs in 185 sequenced genomes of the genus *Pseudomonas* and also compared the protein encoded by the cluster using BLAST analysis against the whole NCBI non-redundant protein database (see Methods for details). This bioinformatics analysis revealed that the complete *gvg* cluster (14 genes) is absent in any of the *Pseudomonas* genomes, apart from the *P*. *fluorescens* WH6 genome (in which it was originally found) (Fig. 4, Table S8). Two strains, *P*. *syringae* pv. *maculicola* strain ES4326 and *P*. *antarctica* strain PAMC 27494, contained genes which matched most of the cluster genes (12 out of 14 genes displayed in 8^th^ and 11^th^ intersection bar from left in Fig. 3, respectively). Both strains (ES4326 and PAMC 27494) harbor all genes involved in the production of the FVG except *gvgB* (EFQ61007.1) gene. The *gvgB* gene codes for a small hypothetical protein and it was exclusively found in WH6. In CDRTc14, only two genes of this cluster were detected, more precisely *gvgK* (EFQ61016.1), a LysE family transporter, which is involved in the transport of the FVG out of the cell^11^, and *ssb* (EFQ61017.1) encoding a ssDNA binding protein. However, the genes involved in FVG production were not found in CDRTc14 (Fig. 4, Table S5). Overall, the genes responsible for FVG production were found very rarely in the *Pseudomonas* genomes investigated, for example only 4.8% of the investigated genomes harboured any gene involved in the production of FVG. Also at the protein level, the products encoded by the *gvg* genes appeared to be very rare (Fig. S8, Table S7). For example, at 60% protein identity threshold, hits (Nhits) for eight FVG producer genes varied between 260 for the *gvgF* gene (EFQ61011.1) to three for *gvgB* (EFQ61007.1). The *gvgB* gene that was not detected in the genus *Pseudomonas* (except WH6) showed similarity with genes found in other genera including *Streptomyces albulus* (62% identity) and *Burkholderia cenocepacia* (89% identity). However, the two FVG transporter genes (*gvgJ* and *gvgK*) were found more commonly showing 449 and 1033 hits at the 60% protein identity threshold, respectively. Overall, it seems that multiple genes of the *gvg* cluster have been horizontally transferred, however, our analysis does not indicate transfer of the complete cluster among the strains/genomes analysed. In particular, the eight genes within the *gvg* cluster supposed to be involved in FVG production occur to be very rare and among them, the small CDS (*gvgB*) consisting of only 28 amino acids seems to be very specific for WH6. FVG belongs to a class of non-proteinogenic amino acids oxyvinylglycine. Some other oxyvinylglycines such as L-2-amino-4-methoxy-trans-3-butenoic acid (AMB), aminoethoxyvinylglycine and rhizobitoxin are also known for their role in plant-microbe interactions but the prediction of their biosynthetic pathways is difficult using bioinformatic tools because their biosynthetic routes might vary considerably between different species^27^. More detailed genetic and metabolomic analysis is needed to obtain further information on the genes involved in the production of oxyvinylglycine or other unknown herbicidal compounds.

Bacteria with herbicidal activity (except WH6) are hardly explored for their herbicidal mechanism at the molecular and genetic level and also bacterial herbicides against broadleaf plants are less often studied than those against grasses^8^. However, auxin-producing bacteria have often been shown effective in inhibiting the growth of broadleaf plants^28,29^. It has been also shown that broadleaf weeds are more prone to certain auxinic herbicides than grassy weeds^30^. In our experiment, we observed that all strains showing herbicidal activity (on broadleaf plants) produced IAA *in vitro* ^31^ (Table S6). Strain CDRTc14 produces indole acetic acid (IAA) up to 42 μg ml^−1^ and harbors genes for IAA production (Table 2). However, different plants generally show different responses to exogenous IAA depending on the concentration perceived by tissues. Rather low levels of IAA stimulate plant growth whereas high concentration of IAA can cause a negative impact on plants^32^. For example, it has been shown that low quantities of IAA produced by *P*. *putida* GR12–2 increased plant growth, while an elevated level of IAA inhibited root growth^33^. Moreover, IAA increases the level of ethylene by stimulating the production of aminocyclopropane-1-carboxylate (ACC) synthase^34^.

**Table 2:** Functional characters tested *in vitro* and their related genes found in CDRTc14.

Functional characters	*In vitro* test	Putative Genes/pathway
HCN	−	*hcnB_1*, *hcnB_2*, *hcnC*
Siderophores	+	*entS*
P solubilization	+	*PqqB*, *PqqC*, *PqqD*, *PqqE*, *PqqF*
IAA	+	indole−3−pyruvate *(IPyA) (iaaM*, *iaaH* gene)
ACC deaminase	−	*acdS*
Antifungal	−	*ubiC*, *gabP*, *gapT*, *gapD*, *PhzF1–2*

Cyanogenic bacteria have been also reported for their herbicidal activity since hydrogen cyanide (HCN) is known to inhibit plant growth by taking part in the metabolism (inhibiting respiratory path, CO_2_ and blocking photosynthetic electron transport) and by finally causing plant death due to cellular hypoxia (cells suffering from lack of oxygen)^35^. CDRTc14 is equipped with genes related to HCN production but did not show HCN activity *in vitro* (Table 2). Bacterial cyanogenesis can be influenced by many factors under laboratory conditions, such as the growth media, cell density, aerobic growth conditions and quorum sensing^36–38^.

### Identification of genes involved in herbicide metabolism and metal resistance

**Figure 5:**
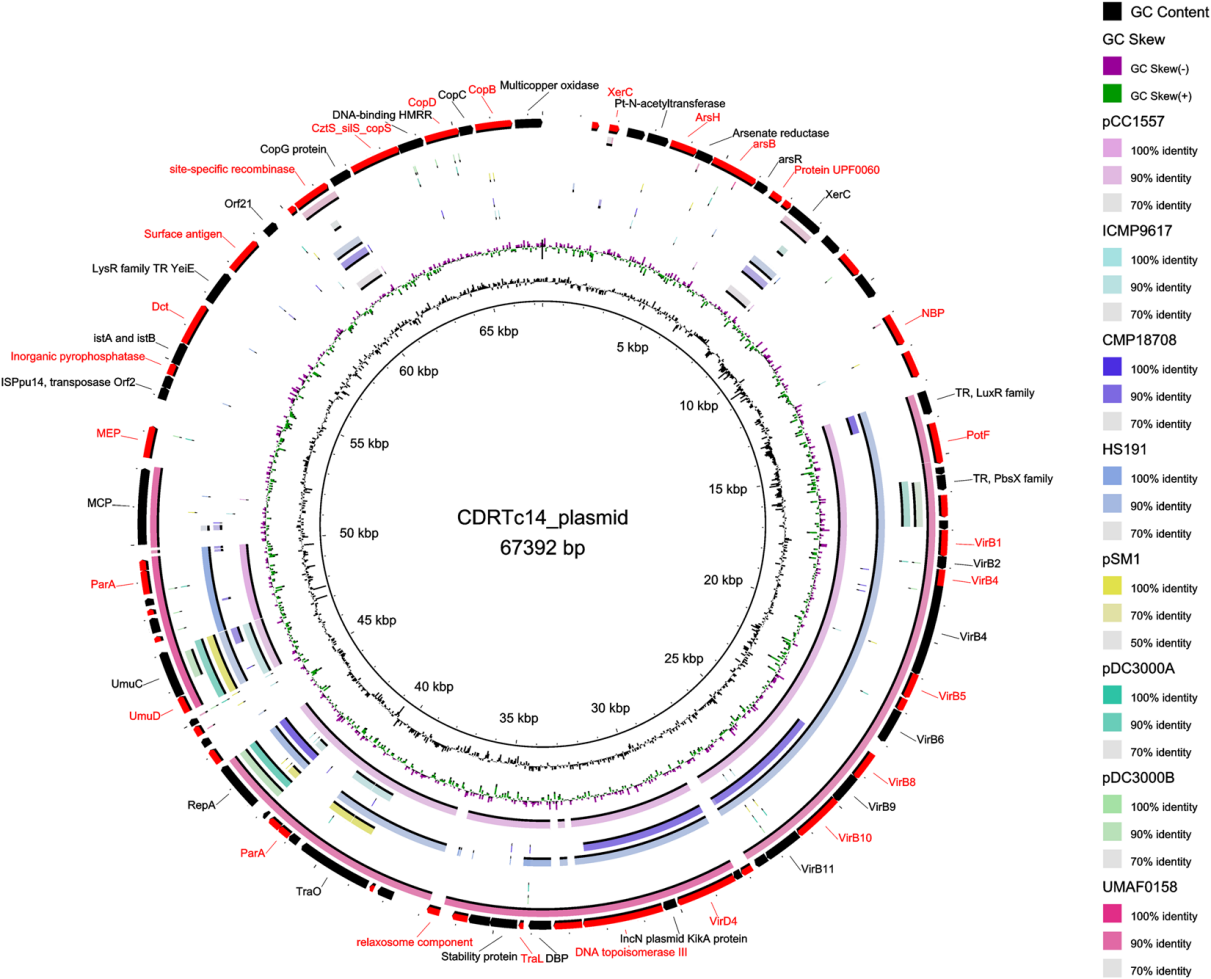
Circular visualization of the comparison of CDRTc14 plasmid with other related (Top 8 blast hits was taken from NCBI using BLASTn, plasmid accession numbers: CP007015.1, NZ_CM002754.1, NZ_CP012180.1, NZ_CP006257.1, CM001987.1, NC_004633.1, NC_004632.1, NZ_CP005971.1). The figure was designed using BRIG. The interesting features are labelled in the outer most circle and annotation is provided in Table S12.

**Figure 6:**
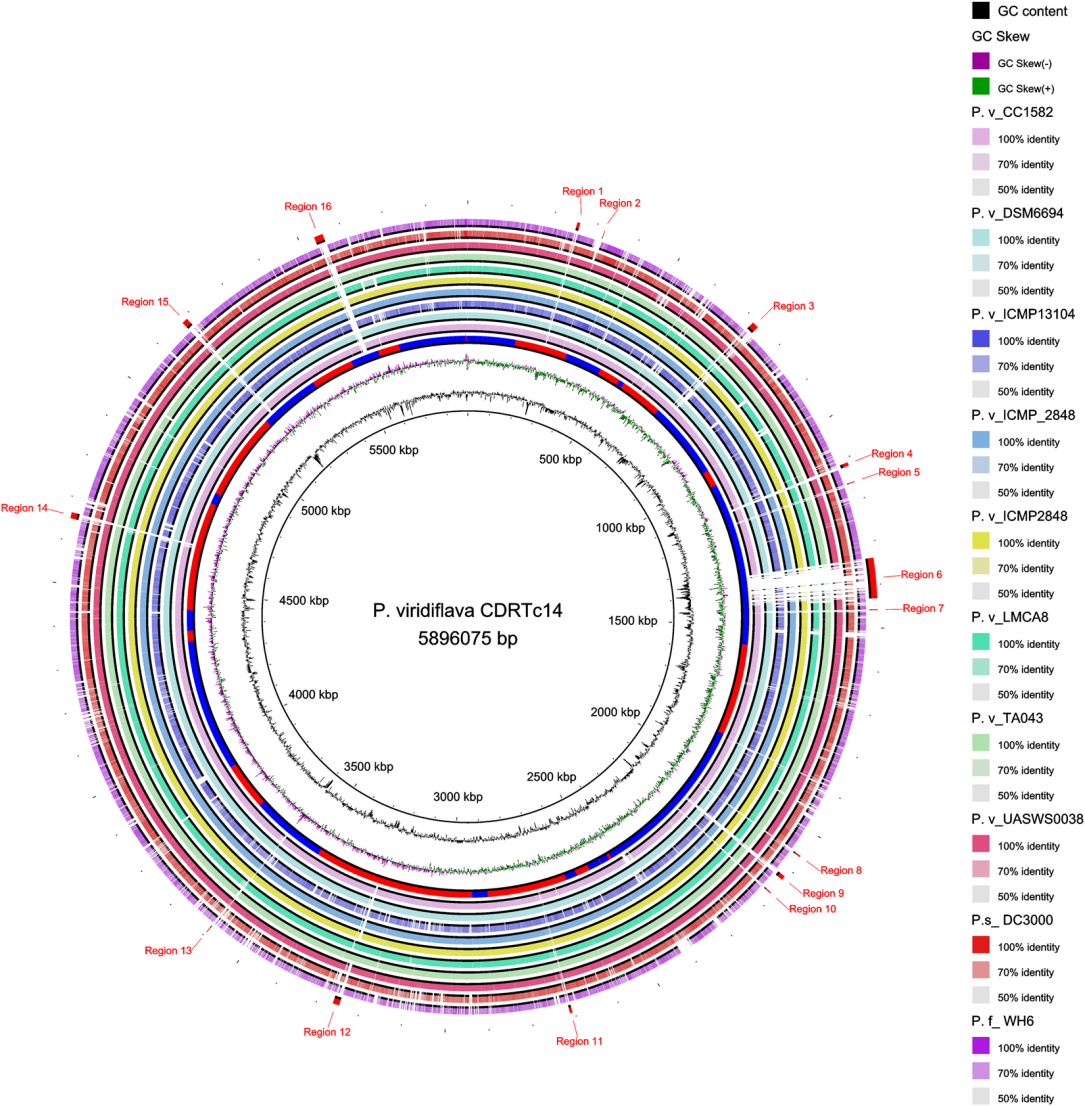
Circular visualization of the whole genome comparison of CDRTc14 chromosome with other related genomes. The figure was designed using BRIG. The gaps in the circles represent regions of low or no similarity provided in Table S11 with annotation.

Plant‐associated bacteria are often described for their potential to degrade commonly used herbicides^39^. In the CDRTc14 genome we found several genes and/or molecular pathways known for the metabolism of commonly used herbicides such as N-(phosphonomethyl) glycine (glyphosate), atrazine, 2,4-dichlorophenoxyacetic acid (2,4 D), alachlor and the complete KEGG pathway of atrazine degradation (predicted by KEGG Automatic Annotation Server) (Table 3). Interestingly, we also found a glufosinate resistance gene (phosphinothricin N-acetyltransferase/RePAT), both on the chromosome and the plasmid of CDRTc14 (Fig. 5 and Table 3). The gene was used to develop glufosinate resistant transgenic rice^40^. *In vitro* testing confirmed (Fig. S9) that CDRTc14 can grow in the presence of glyphosate (20 mM), glufosinate (8 mM), and 2,4 D (1200 mg/L). Strain CDRTc14 could have adapted to herbicides commonly used in vineyards, possibly by acquiring resistance genes through horizontal gene transfer. A wide range of mechanisms for microbial degradation of herbicides has been reported, especially through hydrolytic, bond cleavage, oxidation and reduction reactions^39^. Rhizobacteria and endophytes particularly belonging to the Proteobacteria (α, β and γ classes) have been often described to degrade herbicides and to protect plants from herbicides^41–43^. *P*. *viridiflava* isolates from agricultural fields have also been reported to degrade pesticides^16,17^. Recently, it has been suggested that plant-associated bacteria contribute to herbicide resistance in plants^39^, for example pea (*Pisum sativum* L.) inoculated with an endophytic strain *Pseudomonas putida* POPHV6 showed a higher removal of 2,4 D from soil in the presence of endophytic strain without the translocation of the herbicide into aerial plant parts^44^. Another study in Australia has shown that herbicide resistance of the annual ryegrass (*Lolium rigidum*) is correlated with the colonization of the endophytic fungus *Neotyphodium* spp.^45^. However, the introduction of herbicide resistance traits into plants via microbial engineering has so far been hardly explored. BLAST analysis of the CDRTc14 plasmid and comparison with eight closely related plasmids revealed even more unique features in CDRTc14 (Fig. 6). Many genes found on the CDRTc14 plasmid code for features such as motility, chemotaxis, heavy metal tolerance, herbicidal metabolism (phosphinothricin N-acetyltransferase), DNA repair system, type IV secretion system, and tyrosine recombinase XerC (Fig. 5, Table S12). We also identified putative tyrosine recombinases at the left end of the *RePAT* (phosphinothricin N-acetyltransferase), a well-known herbicide resistance gene. Tyrosine recombinases have been reported to mediate the DNA integration/excision that might be involved in horizontal gene transfer^46^.

**Table 3:** Molecular pathways or genes found in CDRTc14 related to herbicide metabolism.

Herbicide	*In vitro* test	Putative genes/pathway/gene products	Reference
2,4-dichlorophenoxyacetate (2,4-D) and	+	*tfdA* (Alpha-ketoglutarate-dependent 2,4 dichlorophenoxyacetate dioxygenase)	80,81
2-methyl-4-chlorophenoxyacetic (MCPA)	*NA*	*cadA*	77
Atrazine	*NA*	*atzE* (biuret hydrolase), *atzF1–2* (allophanate hydrolase)	82
Glyphosate	+	Enzyme EPSPS (5-enol-pyruvylshikimate-3-phosphate synthase), *aroA1–2 aroA_2* (3-phosphoshikimate 1-carboxyvinyltransferase)argF, sdhA, speA, ahpC (shikimate pathway)	83–85
Glufosinate	+	*bar_1*, *bar_2* (phosphinothricin N-acetyltransferase)	40
Alachlor	*NA*	*gstB_1–5* (glutathione S-transferase))	86

Further, we calculated a pan-genome of all available *P*. *viridiflava* genomes. The *P*. *viridiflava* pan-genome has a total of 11,262 genes: 1,048 represent the core genes (genes present in >99% of isolates within this study), whereas 4,614 and 5,600 form the shell (genes present in 15–95% of isolates analyzed) and cloud genes (genes present in 0–15% isolates analyzed), respectively (Fig. 3A). The genome of DSM_6694 shared the highest number of genes (4,827) while ICMP_13104 shared the least number of genes (1,084) with CDRTc14. Several unique genes (327) were found in the CDRTc14 genome, particularly several genes related to heavy metal resistance including copper (*copB*, *copC*, *copD*) and arsenic (*arsB*, *arsC*, *arsR*, *arsH*) homeostasis, herbicidal resistance (phosphinothricin N-acetyltransferase), type IV secretion system, such as *virB1*, *virB4*, *virB6*, *virB8*, *virB10*, *virB11*, chemotaxis and motility (Table S10). Altogether, comparative analysis between genomes using both BRIG and Roary showed that the CDRTc14 genome is equipped with several unique features located on the chromosome as well on the plasmid. Many of these features correlate with its host *L*. *draba* and the vineyard soil environment. For instance, *L*. *draba* is a noxious weed species, known for the hyperaccumulation of heavy metals^47,48^. It has been shown that metal hyperaccumulating plants host metal-resistant endophytes due to local adaptation to the metal-rich environment^49^. A recent study revealed that long-term association with metal-hyperaccumulator plant leads to local adaptation by *Pseudomonas* endophytes^50^. Copper and arsenic resistant *Pseudomonas* strains have been frequently isolated from the vineyard environment^51,52^. Indeed, vineyards have been treated with copper-containing pesticides to control plant pathogens^53^ as well as with sodium arsenate till the end of the last century, which may still be present in vineyard soils^54^. The fact that CDRTc14 carries heavy metal resistance genes on its plasmid suggests that these genes have been obtained by horizontal gene transfer^53^.

### Identification of genes involved in plant interaction

Endophytes use multiple strategies to interact with their host and their various types of genes have been described for endophytic colonization^55^. A set of known mechanisms, which endophytes often use to interact with plants, are found in the genome of CDRTc14 such as genes coding for type IV pili, motility and chemotaxis (90 genes for flagellar motility, 59 for chemotaxis), transporters (i.e. 80 genes for ABC transporters, 11 for antiporter membrane transporters, six for TRAP transporters, 23 for cation transporters) and protein secretion systems (type I-II & type IV-VII), plant adaptation and protection (i.e. four genes for plant hormones, 15 for siderophores and 93 for stress response), quorum sensing (synthase gene: *acuI*, recipient genes: 14 for autoinducer receptors of LuxR family, *lasR*, *rhtB*, *acuR*) and quorum quenching (*pvdQ*, *quiP*, *aiiA*) (Table S13). To gain in-depth knowledge of the CDRTc14 genome, we performed a comparative genome analysis using all available *P*. *viridiflava* whole genomes (November 11th, 2016), *P*. *syringae* DC3000 and *P*. *fluorescens* WH6 (listed in Table 4). We included *P*. *syringae* DC3000, because it is the most closely related complete genome to CDRTc14 and is a well-studied plant pathogen. Comparison of ten genomes revealed many regions in the CDRTc14 genome showing less (<70%) or no similarity to the other analyzed genomes. These differences are shown in the circular map designed in BRIG (Fig. 6). Interestingly, 16 unique regions were found in CDRTc14 and their annotation revealed 235 coding sequences (CDS) and three coding for RNA (Table S11). Most of these annotated unique regions appear to be similar to the ones predicted by the Roary pipeline during the pan genome analysis (described above in detail).

**Table 4:** General features of the *P*. *viridiflava* CDRTc14 and related organisms used for comparative studies.

Species/strain	Size	% G+C	Scaffolds	Plasmid	Genes	% coding	tRNA	rRNA	GenBank	Isolation source	Location
*P*. *viridiflava* CDRTc14	5.96	59.3	38	1	5,330	97.9	58	9	MBPF00000000	*L*. *draba*	Austria
*P*. *viridiflava* TA043	5.98	59.2	218	—	5,282	98.1	29	2	AVDV00000000	*P*. *officinalis*	France
*P*. *viridiflava* CC1582	6.02	59.2	211	—	5,350	98.0	28	2	AVDW00000000	epilithon	France
*P*. *viridiflava* ICMP2848	5.90	59.4	254	—	5,278	97.0	53	4	LJRS00000000	*P*. *vulgaris*	Switzerland
*P*. *viridiflava* UASWS0038	5.91	59.4	201	—	5,278	97.1	56	10	AMQP00000000	*Rhododendron* sp.	Switzerland
*P*. *viridiflava* LMCA8	5.99	59.3	73	—	5,358	96.9	69	19	JXQO00000000	*A*. *thaliana*	USA
*P*. *viridiflava* DSM 6694	5.91	59.4	188	—	5,256	97.8	54	3	JRXH00000000	*P*. *coccineus*	Switzerland
*P*. *viridiflava* ICMP 13104	5.55	58.9	204	—	4,916	89.4	58	4	LKEJ00000000	*A*. *deliciosa*	France
*P*. *viridiflava* ICMP 2848	5.92	59.4	48	—	5,261	97.8	52	6	LKEH00000000	*P*. *vulgaris*	Switzerland
*P*. *syringae* DC3000	6.4	58.4	1	2	5,721	85.6	15	63	AE016853.1	*S*. *lycopersicum*	UK
*P*. *fluorescens* WH6	6.3	60.6	1	—	5,724	93.8	4	53	CM001025.1	*T*. *aestivum*	USA

Strain CDRTc14 harbors several genes (such as siderophore production, ACC deaminase, and IAA and phosphate solubilization, Table 2) potentially involved in plant growth promotion and conferring enhanced stress tolerance in plants, although under controlled conditions the strain negatively affects its host plant *L*. *draba*. It might be that the herbicidal activity of this strain is bacterial cell-dependent as phytotoxicity can be regulated by quorum sensing^56^. Through quorum sensing bacteria regulate the expression of several phenotypic characteristics such as motility, antibiotics production, phytohormones production in a cell-density dependent manner^56^. Also bacterial autoinducers regulate the expression of virulence genes^57^, a phenomenon which is not restricted to pathogenic interactions. For example, quorum sensing plays a role in the symbiotic interaction between rhizobia and legumes^58^. Furthermore, the expression of virulence genes in a group of (otherwise non-pathogenic) fluorescent pseudomonads has been shown to correlate with their population level inside leatherleaf ferns^59^. Additionally, the amount of IAA produced may reach toxic levels when the population size is high^60^. This correlates with our results obtained in testing dose dependency where we did not observe any negative effect on lettuce growth upon CDRTc14 inoculation with a low inoculum dose (<10^5^ CFU). However, CDRTc14 significantly reduced germination and root length when applied at a higher concentration (CFU = 10^7^) (Figs 1F, S7). Altogether, endophytes may establish very complex and multilayered interactions with their hosts, which range from mutualistic to pathogenic under specific conditions^61^.

### Identification of genes for pathogenicity

**Figure 7:**
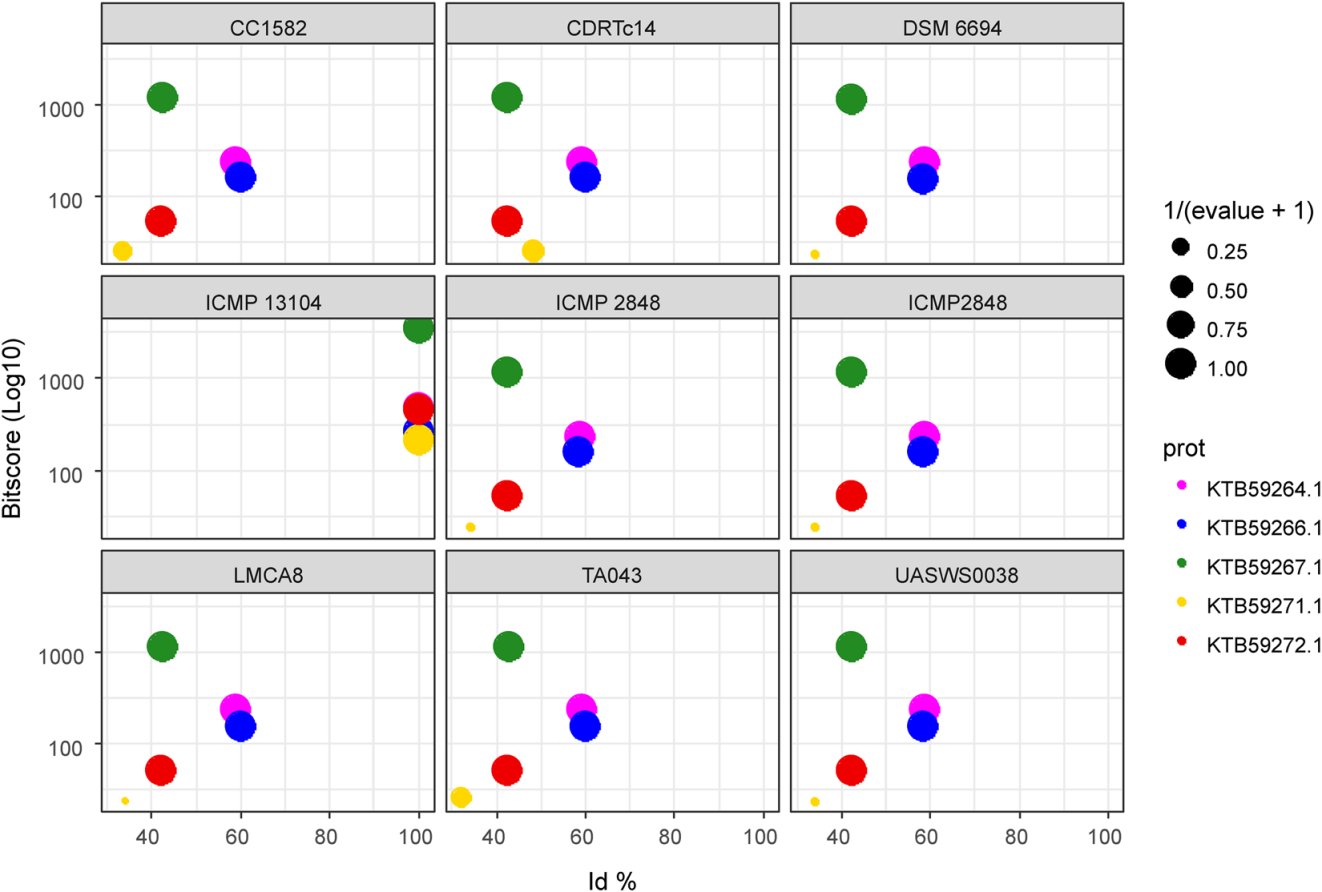
BLAST plot of *P*. *viridiflava* strains tested for the presence of the pathogenicity island of type III secretion system, where strain ICMP 13104 was used as a reference. For this purpose, a tBLASTn search was performed using the gene products of the pathogenicity cluster against the *P*. *viridiflava* genome nucleotide sequences. The scatter plot shows the similarity percentages on the x-axis and the bitscores on the y-axis for each genome, whereas the e-values were transformed as 1/(evalue + 1) so that the size of each point representing a gene product could correlate positively with the e-value significance.

The pathogenicity of the known plant pathogen *P*. *viridiflava* has been correlated with the presence of complete pathogenic islands of type III secretion system^62,63^. We analysed the genomes of all available *P*. *viridiflava* strains using RAST and found altogether seven potential protein secretion systems under membrane transport subsystem category (Table 5). However, the type III secretion system containing thirty genes was only found in strain ICMP 13104, a strain found in kiwi fruits infected with stem cankers (*Actinidia* spp.), while this strain lacks the type I secretion system. In the case of other membrane transport systems, no substantial differences regarding the number of genes were observed among all the strains except for strain ICMP 13104, which was found to be quite different in almost all secretions systems compared to other strains. Furthermore, we screened (using TBLASTN) all *P*. *viridiflava* strains for the presence of the pathogenicity island of type III secretion, using the five pathogenicity related gene products of strain ICMP 13104 as sequence queries. All tested genomes, except ICMP 13104, showed a similarity between 32% and 60% to the five pathogenicity related genes (Figs 7, S7). Moreover, we could not detect the presence of the complete pathogenicity island in CDRTc14 using three different annotation pipelines (PGAAP, Prokka, and RAST). In agreement with this, strain CDRTc14 did not show any pathogenicity symptoms on tomato, *A*. *thaliana*, common bean and *N*. *benthamiana* while other *P*. *syringae* pathovars (DC3000, 1448 A, ATCC11528) showed clearly visible symptoms on all tested hosts species in the greenhouse experiment (Table S3, Fig. S4). Colonization of each inoculated strain was confirmed by 16 S rRNA gene sequencing followed by high resolution melting curve analysis with qPCR (Figs S5 and S6). Moreover, the phylogenetically closest strain (UASWS38) to CDRTc14 also lacks the complete pathogenicity island^25^, whereas strain ICMP 13104 carries the complete pathogenicity island but is phylogenetically only distantly related to CDRTc14 (Fig. 2B). Further research is needed to explore in depth the molecular mechanism underlying virulence-related plant-microbe interactions of CDRTc14.

**Table 5:** *P*. *viridiflava* membrane transport subsystem prediction as estimated by RAST.

Protein family/ Strain (No of total proteins)	CDRTc14	TA043	CC1582	ICMP2848	UASWS0038	LMCA8	DSM 6694	ICMP 13104	ICMP 2848
Protein secretion system, Type I	3	3	3	3	3	3	3	0	3
Protein secretion system, Type II	28	30	30	30	30	30	30	17	30
Protein secretion system, Type III	0	0	0	0	0	0	0	30	0
Protein and nucleoprotein secretion system, Type IV	23	23	23	23	23	23	23	23	23
Protein secretion system, Type V	2	2	2	2	2	3	2	12	2
Protein secretion system, Type VI	40	46	52	41	41	45	40	26	40
Protein secretion system, Type VII (Chaperone/Usher pathway, CU)	1	1	1	1	1	1	1	1	1
ABC transporters	80	84	83	80	79	76	79	73	79
Uni- Sym- and Antiporters	11	11	11	11	11	11	11	10	11
Membrane Transport - no subcategory	54	59	59	59	58	54	59	41	59
TRAP transporters	6	6	6	6	6	6	6	3	6
Protein translocation across cytoplasmic membrane	7	7	6	7	7	8	7	7	7
Cation transporters	23	18	18	19	18	19	19	15	19

## Conclusions

Functional assays in combination with an in-depth comparative genome analysis revealed that strain CDRTc14, which was isolated as a root endophyte from a perennial weed in a vineyard, can be assigned to *P*. *viridiflava*. Although this species comprises many plant pathogens strain CDRTc14 did not induce disease symptoms on the plant species tested in this study and lacks known virulence genes. Moreover, strain CDRTc14 shows a number of features, which are well known from beneficial plant-associated bacteria. However, this strain showed herbicidal activity against its host and to other related plant species, while it showed neutral or beneficial effects on the growth of others. These findings refute the centrifugal phylogenetic method (CPM), which is used as a standard in host-specificity testing of biological control agents. Strain CDRTc14 shows similar herbicidal activities as strain WH6, but the gene cluster responsible for herbicidal activity in this strain was absent in CDRTc14, suggesting that both strains employ different mechanisms. Although genetic and metabolomics analyses are needed to further elucidate the mechanisms responsible for and metabolites involved in herbicidal activity, we found activities and responsible genes indicating the adaptation of this strain to conditions typically encountered in vineyards such as heavy metal resistance and herbicide/pesticide degradation. The amount of pesticide application in vineyards is in comparison to other crops extremely high. Furthermore, pesticides based on copper are still widely used and products with arsenic were used in the past. Features like herbicide degradation and heavy metal resistance reflect the adaptation of strain to prevailing soil environmental stressors.

## Methods

### Germination assay in growth chamber

Germination tests were carried out to evaluate the herbicidal effect of bacterial inoculation on different plant species. All isolates used in this assay were previously isolated from a vineyard field (*L*. *draba* or grapevine)^31^ and CDRTc14 was isolated from surface sterilized roots of the vineyard weed *L*. *draba* L.^31^ (Table S6). The seeds of all tested plants (*L*. *draba* lettuce and *A*. *thaliana* Col-1) were surface sterilized by immersion in 3.25% (v/v) sodium hypochlorite (NaOCl) for 1 min, followed by 70% (v/v) ethanol for 1 min, rinsed five times in sterile distilled water and blotted on sterilized filter paper. Bacterial cultures, grown for overnight at 28 °C in R2A broth (Lab M limited, Lancashire, United Kingdom), were adjusted to an OD_600_ of 0.2 (∼10^7^ CFU ml^−1^). Then 10 ml of each bacterial culture were centrifuged at 4.000 x g for 20 min at 10 °C and after discarding the supernatants bacterial pellets were dissolved into 10 ml PBS (phosphate-buffered saline). Five ml of the bacterial culture was used for seed imbibement for 30 min. Each treatment contained three replicates, for a mock treatment PBS alone was used. Fifteen to twenty surface-sterilized seeds were plated on a 145 mm Petri dish containing approximately 60 mL of 1% water agar medium and placed in a plant culturing room under conditions of 16/8 hours of day/night (20 °C, 50% air humidity) for 12–18 days depending on the growth of the various plant species.

### Greenhouse experiment with *Lepidium draba*

Six isolates (Table S6) were further tested on *L*. *draba* under greenhouse conditions (an average temperature of 29 °C, a light regime of 12:12 h L: D (light: dark), and 68–80% relative humidity). In the greenhouse high pressure sodium lamp with clear outer bulb (MASTER Agro 400 W E40 1SL/12, Philips) was used as a source of light energy. Ten seeds of *L*. *draba* were sown in small plastic pots (500 g substrate containing 3 parts Einheitserde classic and 1 part premium perlite of Gramoflor, Germany) in triplicates for each treatment. Bacterial pellets from five ml of each bacterial culture (OD_600_ = 0.2) were resuspended in PBS and dispensed onto seeds in the pots just after sowing, and control was treated with 5 ml PBS (pH = 7.4). Plants were harvested after 9 weeks. Germination percentage, root length, shoots length and fresh biomass of seedlings were recorded.

### Plant host range test of CDRTc14

To test the host range of strain CDRTc14, seven different plant species, closest relative of *L*. *draba* (*Lepidium meyenii*, *Hesperis matronalis*, *Isatis tinctoria*, *Erysimum odoratum*, *Sisymbrium officinale*, *Lepidium sativum*, *Brassica n*. var. Cleopatra, *B*. *napus* var. Pharao) (Fig. S3) were tested under greenhouse conditions using the same protocol as mentioned above, but instead of pots 145 mm petri dishes were used (with 10 seeds per petri dish). Plants were harvested after 3–4 weeks depending on growth of different plant species. The number of seeds germinated was counted daily, root and shoot length of all the germinated seedlings were recorded in cm and fresh biomass of all seedlings were recorded in g.

### Dose dependent test of CDRTc14 on lettuce

To evaluate if the phytotoxicity of CDRTc14 is concentration dependent we tested 10^1^ to 10^7^ CFU ml^−1^ of CDRTc14 on lettuce seeds. Lettuce was chosen due to its faster and higher germination rate than *L*. *draba*. All the experimental conditions were the same as described above for growth chamber experiment with the exception of plate type and number of replicates, 12 wells plates were used instead of 145 mm Petri dish and each treatment contained five replicates.

### Genome sequencing, annotation and comparative analysis

The information about the CDRTc14 whole genome sequencing, genome assembly and quality check was reported earlier^16^. The CDRTc14 genome was annotated using the NCBI Prokaryotic Genome Automatic Annotation Pipeline (PGAAP) as well as Prokka^64,65^. RAST (Rapid Annotation using Subsystem Technology) was used to compare different *P*. *viridiflava* strains at the genome level^66^.

All genomes used in this analysis were downloaded in FASTA format using the NCBI-genome-download python script (available at https://github.com/kblin/ncbi-genome-download). The *gvg* biosynthetic gene cluster products were downloaded in FASTA format using the NCBI E-utils (available at ftp://ftP.ncbi.nlm.nih.gov/entrez/entrezdirect/). Then an Average Nucleotide Analysis (ANI) was calculated using the pyani Python3 module (available at https://github.com/widdowquinn/pyani) with both MUMmer (NUCmer) alignment and BLAST search methods used as confirmation.

Pangenome analysis of all strains was performed with Roary^67^ after genome annotation in Prokka. Functional annotation of core genes of CDRTc14 and WH6 was performed using the EggNOG 4.5 resource^68^. Genome wide search for the *gvg* genes cluster was done by adopting two different approaches: (1) a tBLASTn search of each gene of the *gvg* cluster against the whole set of *Pseudomonas* complete genomes available in NCBI (November 11^th^, 2016) including also four draft genomes of *P*. *viridiflava* CDRTc14, *P*. *viridiflava* UASWS0038, *P*. *syringae* ES4326 and *P*. *fluorescens* WH6. The best hit of each gene against each nucleotide genome sequence was used to generate a table reporting bitscores, similarity percentages and e-values. The table was processed further and binary values generated assuming 75% of sequence similarity as a threshold for putative homology. The table was imported in R and an UpSet technique^69^ applied using the UpSetR package. (2) A BLASTp search was conducted for each *gvg* cluster gene product against the entire NCBI nr database (November 25th, 2016) in R, using the Bio3D package^70^. Normalized scores were clustered to partition hits in groups by similarity to query and e-value, bitscore, identity and length values of the hits showed in a plot for each accession. BLAST Ring Image Generator (BRIG) was used for visualisation of genome comparisons^71^ and bacterial annotation system (BASys) was used to prepare CDRTc14 circular image with annotation^72^.

### Pathogenicity test

Plant pathogenicity tests were performed in the greenhouse to test compatible (disease) and incompatible (resistance) responses due to strain CDRTc14 on tomato (var. Cobra), *A*. *thaliana* (Col-0), common bean (var. Roma II) and *Nicotiana benthamiana* which are known susceptible host of *P*. *syringae* ^73,74^ and *P*. *viridiflava* ^13,75^. Bacterial cultures (OD_600_ = 0.2) in PBS buffer with 0.01% Silwet L-77 (OSI Specialties Inc., Danbury, CT, U.S.A.) were sprayed to runoff on the abaxial and adaxial leaf surfaces of about 3-week-old plants. Control plants were treated with PBS buffer containing 0.01% Silwet L-77. *N*. *benthamiana* plants were also infiltrated with a needleless syringe to find out if cells produce a hypersensitive response. The pathogenicity test was based on a completely randomized design with three replications in the greenhouse conditions as mentioned above. The plants were scored for bacterial speck severity one week after inoculation using the scale of 0–3 (0 = free of symptoms). Pathogenic strains of each plant species were used as reference and are listed in Table S3.

### Re-isolation of bacteria from plant tissues and high resolution melting PCR (HRMA)

Leaves were detached 3 weeks after the inoculation and surface sterilized (70% ethanol for 30 s, followed by rinsing with sterile distilled water 3 times). The surface sterilization was checked by plating the last washing on R2A. Leaf discs from three different leaves were ground in 10 mM MgCl_2_ with a glass rod into test tubes. Then the samples were thoroughly vortex-mixed and 1:10 serially diluted. Samples were plated on King’s B medium and incubated at 28 °C for 2 days. Resulting colonies were picked and subjected to high resolution melting analysis (HRMA)^76^. Pure cultures of each reference strain (DC3000, 1448 A, ATCC11528, listed in Table S3) was also run in parallel for melting curve comparison. The PCR was carried out on a CFX96 cycler (Bio-Rad Laboratories, Inc., Hercules, CA, USA), with 10 µl reaction mixture containing 0.5 µl (10 µM) of each primer (Gamma395F 5′-CMATGCCGCGTGTGTGAA-3′ and Gamma871r 5′-ACTCCCCAGGCGGTCDACTTA-3′) targeting Gammaproteobacteria^77^, 5 µl of 2 × SsoFast EvaGreen Supermix (Bio-Rad Laboratories, Inc.), 3 µl water and 1 µl (10 ng) genomic DNA. Cycling conditions were one cycle at 95 °C for 5 min, followed by 40 cycles at 95 °C for 5 s and 60° for 5 s. Melting curves of PCR amplicons were obtained with temperatures increasing from 65 °C to 95 °C. Each single DNA batch was done in triplicates, and analyzed by high-resolution melting analysis software (Bio-Rad Laboratories, Inc.), which automatically clusters the samples per their melting profiles and assigns a confidence score to each sample (Fig. S5). Pure genomic DNA of each inoculated strain was used as reference to confirm the identity of each sample tested by comparing its melting profile to the reference. The confidence level threshold for a sample to be included in a cluster was 99.5%. The HRM data were confirmed by sequencing the amplicons of each cluster using the Gamma395F primer and phylogenetic analysis was performed using MEGA6^78^.

### Herbicide resistance assay

CDRTc14 was grown in R2A broth (Lab M, UK) at 28 °C for overnight. The culture was collected by centrifugation and washed twice using liquid M9 minimal medium^79^. Bacteria in the culture (OD_600_ = 0.2) were then grown with shaking at 28 °C in liquid M9 minimal medium supplemented with either glyphosate (at the concentrations of 0, 5, 10, 15, 20 mM), glufosinate (2, 4, 6, 8 mM) or 2, 4 D (at the concentrations of 200, 400, 800 and 1200 mg/l). The OD_600_ values of the cultures were determined at 1-h intervals to record the growth rates of the strains till 48 h. Alternatively, bacteria were plated on M9 agar^79^ containing herbicide as mentioned above. After 5 days of incubation at 28 °C, the colonies growth was observed on the plates.

### Availability of data

The datasets generated during and/or analysed during the current study are available from the corresponding author on reasonable request.

## Electronic supplementary material

Supplementary Material and Methods and Figures

Supplementary Tables S8-S13
