# Supplementary material for: Comparative genome analysis of the vineyard weed endophyte Pseudomonas viridiflava CDRTc14 showing selective herbicidal activity
Source: Sci Rep. 2017 Dec 11;7:17336. doi: 10.1038/s41598-017-16495-y (PMC5725424; doi:10.1038/s41598-017-16495-y)

Supplementary Materials

**Comparative genome analysis of the vineyard weed endophyte *Pseudomonas viridiflava* CDRTc14 showing selective herbicidal activity**

Abdul Samad^1^, Livio Antonielli^1^, Angela Sessitsch^1^, Stéphane Compant^1^ & Friederike Trognitz^1^

^1^ AIT Austrian Institute of Technology, Center for Health and Bioresources, Bioresources Unit, Konrad-Lorenz-Straße 24, A-3430 Tulln, Austria

Running title: Comparative genomic analysis of herbicidal bacterium *Pseudomonas viridiflava* CDRTc14

Corresponding author: friederike.trognitz@ait.ac.at

**Supplementary Tables**

**Table S1.** Genome sequencing project information.

| **Property** | **Term** |
| --- | --- |
| Finishing quality | Draft |
| Libraries used | Illumina paired-end library |
| Sequencing platforms | Illumina, Hiseq |
| Fold coverage | 221.37X ± 55.51 |
| Assemblers | SPAdes 3.8.0 |
| Gene calling method | GeneMarkS+ (PGAAP); Prodigal 2.60 (Prokka 1.11) |
| Locus Tag | BB779 |
| Genbank ID | MBPF00000000 |
| GenBank Date of Release | 29/08/2016 |
| BIOPROJECT | PRJNA329466 |
| Source Material Identifier | CDRTc14 |
| Project relevance | Genome comparison |

**Table S2. General genome features of *Pseudomonas viridiflava* strain CDRTC14**

| Feature | Chromosome | Plasmid | Total |
| --- | --- | --- | --- |
| Length (bp) | 5,896,075 | 67,392 | 5,963,467 |
| G + C content (%) | 59.3 | 55 | 59.3 |
| DNA scaffolds | 37 | 1 | 38 |
| Total genes | 5,358 | 79 | 5,437 |
| Protein coding genes | 5,183 | 66 | 5,249 |
| rRNA number | 9 | 0 | 9 |
| tRNA number | 58 | 0 | 58 |
| Others | 121 | 0 | 4 |
| GenBank accession | - | MBPF00000000(NODE_23) | MBPF00000000 |

Table S3. Bacterial strains and plant species used in the pathogenicity experiment

| Pathovar | Strain | Disease | Hosts | Cultivar | Collection Source |
| --- | --- | --- | --- | --- | --- |
| *P. syringae* pv. tomato | DC3000 (NCPPB 4369) | bacterial speck | tomato | Cobra | BOKU, Austria |
| *P. syringae* pv. tomato | DC3000 (NCPPB 4369) | bacterial speck | *Arabidopsis* | Col-1 | BOKU, Austria |
| *P. syringae* pv. phaseolicola | 1448A (NCPPB 4478) race 6 | halo blight | green bean | Roma II | DSMZ, Germany |
| *P. syringae* pv. tabaci | ATCC11528 | wildfire | *Nicotiana benthamiana* |  | AIT, Austria |

**Table S4.** *P. viridiflava* subsystem prediction as estimated by using RAST.

| **Protein family/ Strain (No of total proteins)** | **CDRTc14** | **TA043** | **CC1582** | **ICMP2848** | **UASWS0038** | **LMCA8** | **DSM 6694** | **ICMP 13104** | **ICMP 2848** |
| --- | --- | --- | --- | --- | --- | --- | --- | --- | --- |
| Co-factors, Vitamins, Prosthetic Groups, Pigments | 334 | 331 | 331 | 332 | 333 | 326 | 332 | 320 | 332 |
| Cell Wall and Capsule | 203 | 204 | 204 | 197 | 199 | 202 | 197 | 183 | 197 |
| Virulence, Disease and Defense | 133 | 123 | 123 | 132 | 126 | 127 | 130 | 109 | 130 |
| Potassium metabolism | 23 | 23 | 20 | 23 | 23 | 23 | 23 | 26 | 23 |
| Photosynthesis | 0 | 0 | 0 | 0 | 0 | 0 | 0 | 0 | 0 |
| Miscellaneous | 46 | 45 | 45 | 46 | 45 | 46 | 45 | 45 | 45 |
| Phages, Prophages, Transposable elements, Plasmids | 6 | 10 | 20 | 15 | 14 | 32 | 14 | 8 | 14 |
| Membrane Transport | 278 | 290 | 294 | 282 | 279 | 279 | 280 | 258 | 280 |
| Iron acquisition and metabolism | 43 | 54 | 53 | 47 | 45 | 46 | 47 | 47 | 46 |
| RNA Metabolism | 212 | 214 | 212 | 212 | 212 | 210 | 211 | 212 | 211 |
| Nucleosides and Nucleotides | 120 | 119 | 119 | 120 | 115 | 120 | 120 | 111 | 120 |
| Protein Metabolism | 282 | 261 | 265 | 279 | 282 | 284 | 280 | 285 | 278 |
| Cell Division and Cell Cycle | 33 | 33 | 33 | 33 | 33 | 34 | 33 | 37 | 33 |
| Motility and Chemotaxis | 149 | 147 | 149 | 146 | 144 | 150 | 144 | 154 | 144 |
| Regulation and Cell signaling | 117 | 107 | 114 | 85 | 108 | 113 | 108 | 111 | 108 |
| Secondary Metabolism | 5 | 5 | 5 | 5 | 5 | 5 | 5 | 5 | 5 |
| DNA Metabolism | 119 | 105 | 109 | 110 | 109 | 109 | 109 | 113 | 109 |
| Fatty Acids, Lipids, and Isoprenoids | 193 | 200 | 197 | 196 | 197 | 167 | 196 | 175 | 199 |
| Nitrogen Metabolism | 40 | 40 | 39 | 42 | 41 | 41 | 41 | 46 | 41 |
| Dormancy and Sporulation | 4 | 4 | 5 | 4 | 5 | 4 | 4 | 5 | 4 |
| Respiration | 123 | 122 | 123 | 125 | 125 | 128 | 125 | 103 | 125 |
| Stress Response | 193 | 195 | 196 | 195 | 200 | 194 | 194 | 184 | 194 |
| Metabolism of Aromatic Compounds | 80 | 80 | 79 | 79 | 79 | 80 | 79 | 73 | 80 |
| Amino Acids and Derivatives | 552 | 565 | 556 | 554 | 559 | 556 | 552 | 546 | 558 |
| Sulfur Metabolism | 90 | 90 | 96 | 91 | 97 | 90 | 91 | 86 | 91 |
| Phosphorus Metabolism | 54 | 52 | 52 | 54 | 54 | 52 | 54 | 55 | 54 |
| Carbohydrate | 421 | 423 | 423 | 419 | 415 | 420 | 418 | 415 | 416 |

**Table S5.** Similarity of FVG biosynthetic genes of WH6 with CDRTc14

| **GenBank accesion** | **Gene locus PFWH6** | **Gene** | **Putative function** | **Nucleotide similarity with CDRTc14** | **Amino acid Similarity with CDRTc14** |
| --- | --- | --- | --- | --- | --- |
| EFQ61004.1 | PFWH6_5247 | *tam* | Trans-aconitate methyltransferase | 29.17% | 28/96 (29%) |
| EFQ61005.1 | PFWH6_5248 | *gvgR* | GntR-family transcriptional regulator | 66.74 | 313/469 (66%) |
| EFQ61006.1 | PFWH6_5249 | *gvgA* | Esterase/lipase domain-containing protein | 0 | 20/75 (26%) |
| EFQ61007.1 | PFWH6_5250 | *gvgB* | Hypothetical small protein | 0 | 11/28 (39%) |
| EFQ61008.1 | PFWH6_5251 | *gvgC* | Heme-oxygenase domain-containing protein | 32.24 | 59/183 (32%) |
| EFQ61009.1 | PFWH6_5252 | *gvgD* | Amidinotransferase | 0 | 22/68 (32%) |
| EFQ61010.1 | PFWH6_5253 | *gvgE* | LysE Family Transporter | 50.29 | 91/182 (50%) |
| EFQ61011.1 | PFWH6_5254 | *gvgF* | Carbamoyltransferase | 29.93 | 183/608 (30%) |
| EFQ61012.1 | PFWH6_5255 | *gvgG* | Hypothetical small protein | 0 | 10/35 (28%) |
| EFQ61013.1 | PFWH6_5256 | *gvgH* | Aminotransferase | 29.86 | 129/430 (30%) |
| EFQ61014.1 | PFWH6_5257 | *gvgI* | Formyltransferase | 26.99 | 44/163 (26%) |
| EFQ61015.1 | PFWH6_5258 | *gvgJ* | LysE Family Transporter | 28.85 | 60/208 (28%) |
| EFQ61016.1 | PFWH6_5259 | *gvgK* | LysE Family Transporter | 76.73 | 155/202 (76%) |
| EFQ61017.1 | PFWH6_5260 | *ssb* | ssDNA binding protein | 77.72 | 137/190 (72%) |

**Table S6**. Six bacterial isolates with herbicidal activity and their tested functional characteristics *in vitro*.

| Isolate ID | Isolation source | Closest relative (16S rRNA gene) | Similarity % | Functional characteristics | | | | | |
| --- | --- | --- | --- | --- | --- | --- | --- | --- | --- |
|  |  |  |  | HCN | Siderophore | Phosphate Solublization | IAA | ACC deaminase | Antifungal |
| CDRTc14 | *L. draba* root | *P. viridiflava* | 100% | - | + | + | + | - | - |
| VVRTb5 | Grapevine root | *Bosea* sp. *T10-3D-2C* | 99% | - | - | - | + | - | - |
| VVRTb6 | Grapevine root | *Rhizobium* sp. *SWFU-R27* | 99% | - | - | + | + | + | - |
| VVRe17 | Grapevine rhizosphere | *Arthrobacter* sp. *strain CSR_5* | 99% | - | - | - | + | + | - |
| CDRb3 | *L. draba* rhizosphere | *Agromyces humatus* | 98% | - | - | - | + | - | - |
| CDRTb13 | *L. draba* root | *P. fluorescens* | 99% | + | - | + | + | - | - |

**Table S7.** Summary of NCBI BLAST protein sequence search (blast.pdb) for of FVG biosynthetic genes of WH6 across entire NCBI protein database

| **Protein accession no.** | **gene** | **>80%** | **60-80%** | **40-60%** | **Total** |
| --- | --- | --- | --- | --- | --- |
| EFQ61004.1 | *tam* | 200 | 0 | 0 | 200 |
| EFQ61005.1 | *gvgR* | 32 | 159 | 0 | 191 |
| EFQ61006.1 | *gvgA* | 26 | 43 | 332 | 401 |
| EFQ61007.1 | *gvgB* | 2 | 1 | 0 | 3 |
| EFQ61008.1 | *gvgC* | 21 | 37 | 160 | 218 |
| EFQ61009.1 | *gvgD* | 49 | 18 | 107 | 174 |
| EFQ61010.1 | *gvgE* | 30 | 27 | 144 | 201 |
| EFQ61011.1 | *gvgF* | 50 | 210 | 0 | 260 |
| EFQ61012.1 | *gvgG* | 5 | 12 | 7 | 24 |
| EFQ61013.1 | *gvgH* | 54 | 14 | 163 | 231 |
| EFQ61014.1 | *gvgI* | 56 | 4 | 118 | 178 |
| EFQ61015.1 | *gvgJ* | 37 | 412 | 3 | 452 |
| EFQ61016.1 | *gvgK* | 33 | 1033 | 0 | 1066 |
| EFQ61017.1 | *ssb* | 302 | 40 | 31 | 373 |

**Supplementary Tables S8-S13 provided in Excel format.**

**Table S8.** Results of NCBI BLAST nucleotide sequence search (blast.pdb) for FVG biosynthetic genes of WH6 (*gvg* gene cluster) across all available complete *pseudomonas* genomes.

**Table S9.** Annotation of core genes of two herbicidal strains, CDRTc14 and WH6.

**Table S10.** Annotation of core and unique genes of all *P. viridiflava* strains analysed for pangenome.

**Table. S11.** Annotation of low similarity regions/unique regions found in CDRTc14 chromosome compared to the genomes of other tested strains in the study.

**Table S12.** Annotation of low similarity regions/unique regions found in CDRTc14 plasmid compared to the plasmid of other tested strains in the study.

**Table S13.** Genes in CDRTc14 genome related to plant-associated/endophytic life style.

**Figure Legends**

Fig. S1. *In vitro* plant bioassay: (A) Effect of bacterial inoculation on seeds germination (B) effect of bacterial inoculation on radicle length of seedlings, PBS (phosphate-buffered saline) used as control. Details about the isolates are provided in Table. S6.

Fig. S2. Dose dependent effect of CDRTc14 on lettuce. (A) 10^1^-10^5^ CFU was used for inoculation of lettuce seeds (B) 10^5^-10^7^ CFU ml^-1^ was used for inoculation of lettuce seeds (C) root length of the seedlings (D) seedling biomass

Fig. S3 Phylogeny of plant species used in host range test of CDRTc14. The tree is generated in MEGA 6 using chloroplast *ndhF* gene (1987bp) from NCBI database.

Fig. S4. Disease symptoms on plant leaves, seven days after spray-inoculation with bacterial suspension (10^7^ CFU ml^-1^ containing 0.01% silwet L-77). Where A, D, G and J were spray inoculated with their respective pathovar (provided in Table S1), B, E, Hand K were spray inoculated with CDRTc14 and C, F, I and L were inoculated with PBS containing 0.01% silwet (phosphate-buffered saline).

Figure S5. High resolution melting (HRM) analysis for discrimination of different *P. syringae* pathovar and CDRTc14. Normalized and difference plots obtained in multiplex HRM assay. Pure genomic DNAs from each isolate was used as control. On each plot, different colors indicate distinct profiles for each isolate. Green and red columns represent pre- and post-melting normalization regions.

Fig. S6. Phylogenetic tree of bacteria re-isolated from surface sterilized leave of green bean, tomato, *N. benthamiana* and *Arabidopsis*, two weeks after spray inoculation. 16S rRNA amplification was done on qPCR followed by high resolution melting analysis (HRMA) and subsequently partial 16S rRNA region was sequenced.

Fig. S7. Whole genome comparison of CDRTc14 with *P. viridiflava* ICMP 13104 showing very low similarity for Type III Secretion system. The figure was designed using BRIG.

Fig. S8 (A-N) BLAST Results of FVG biosynthetic genes of WH6 (with blast.pdb function) at protein level across entire NCBI protein database (nr) using bio3d R package. Results are visualized and filtered by using function plot.blast where top score hits are indicated in black color. Blast hit results (Nhit) results are summarized in Table S7.

Fig. S9 Growth kinetics of CDRTc14 in liquid M9 minimal medium supplemented with herbicide, (A) glyphosate (B) glufosinate (C) 2, 4 D. Values are the mean of triplicate assays ± SD.

Fig. S1


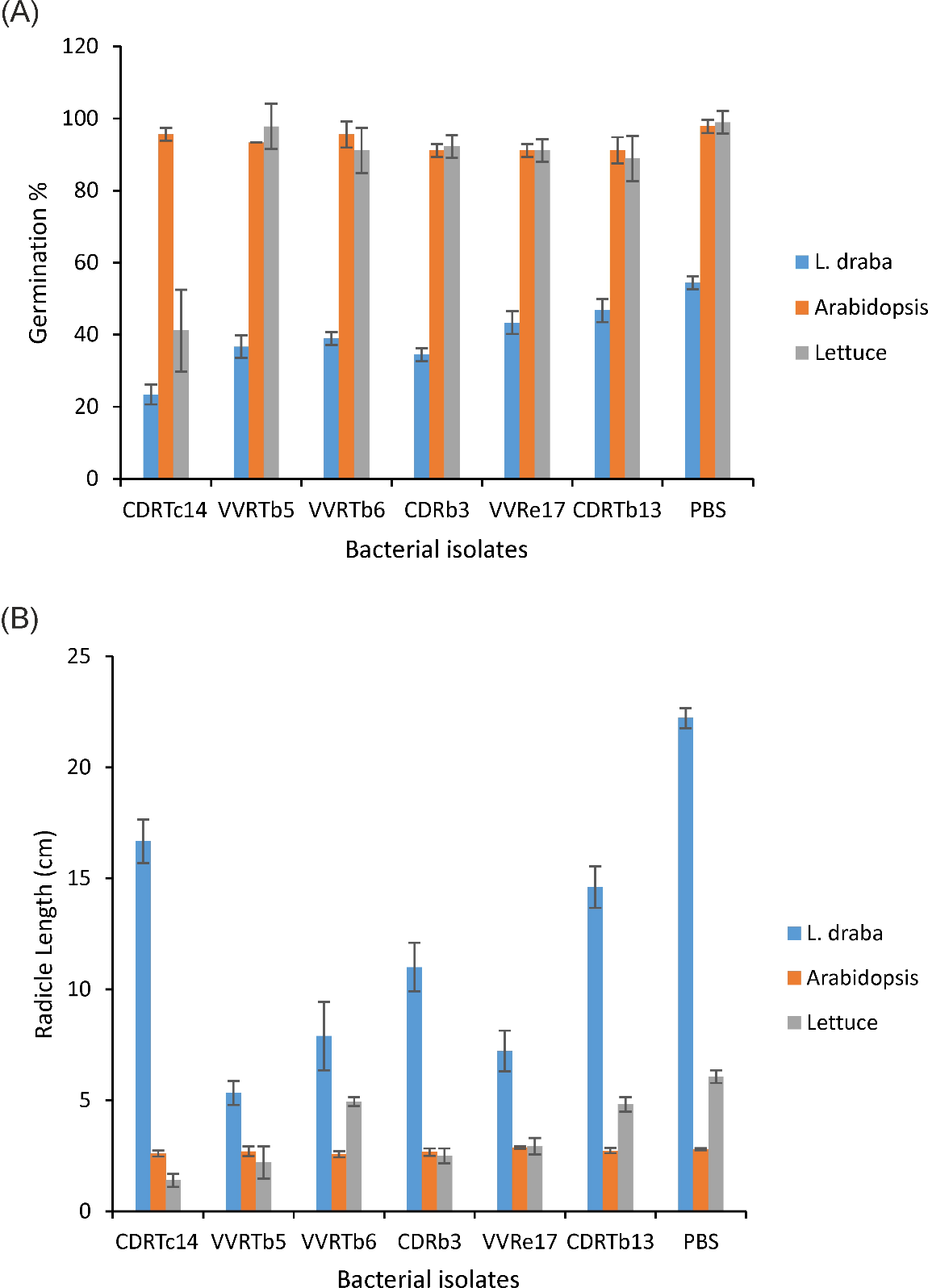


Fig. S2


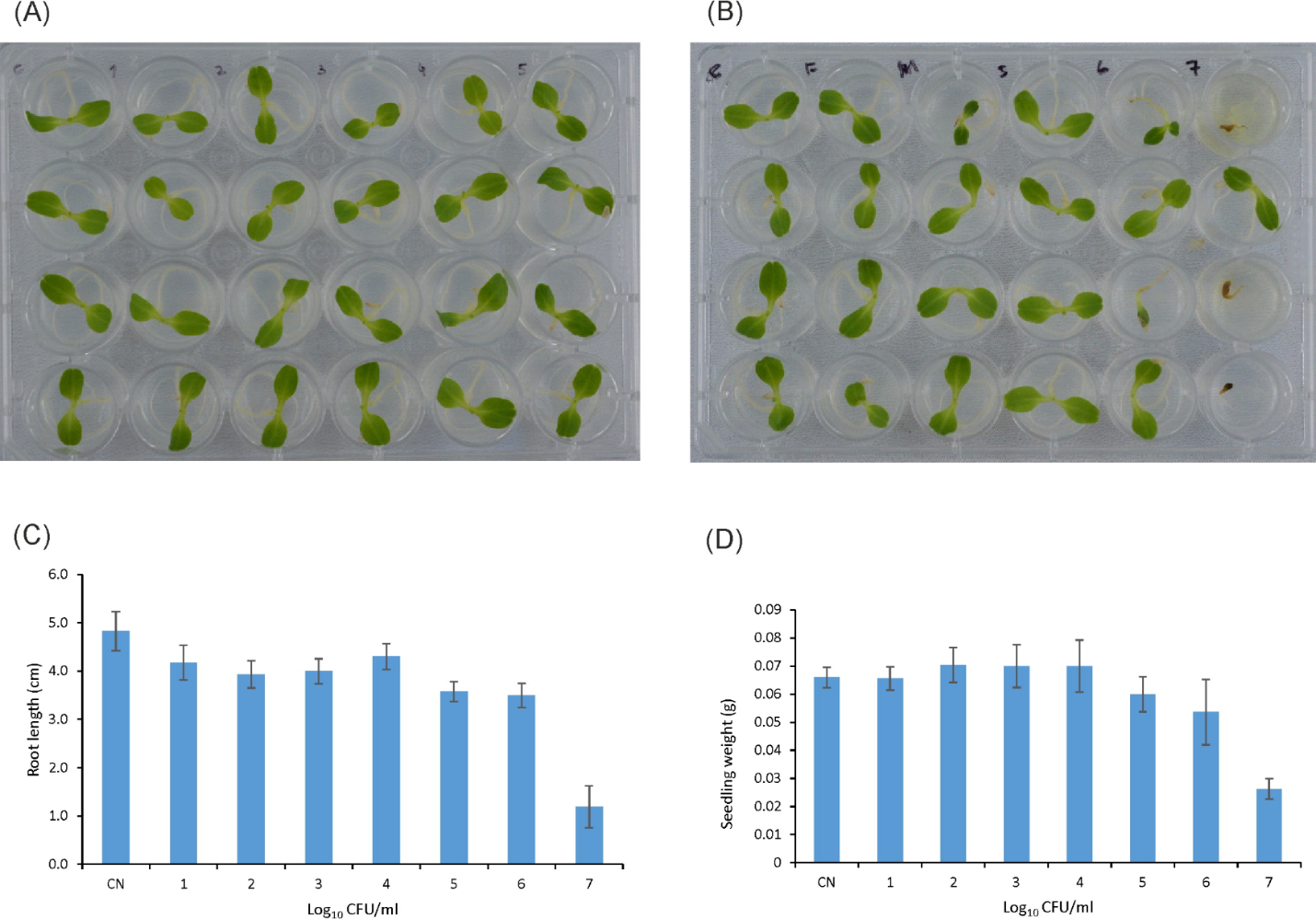


Fig. S3


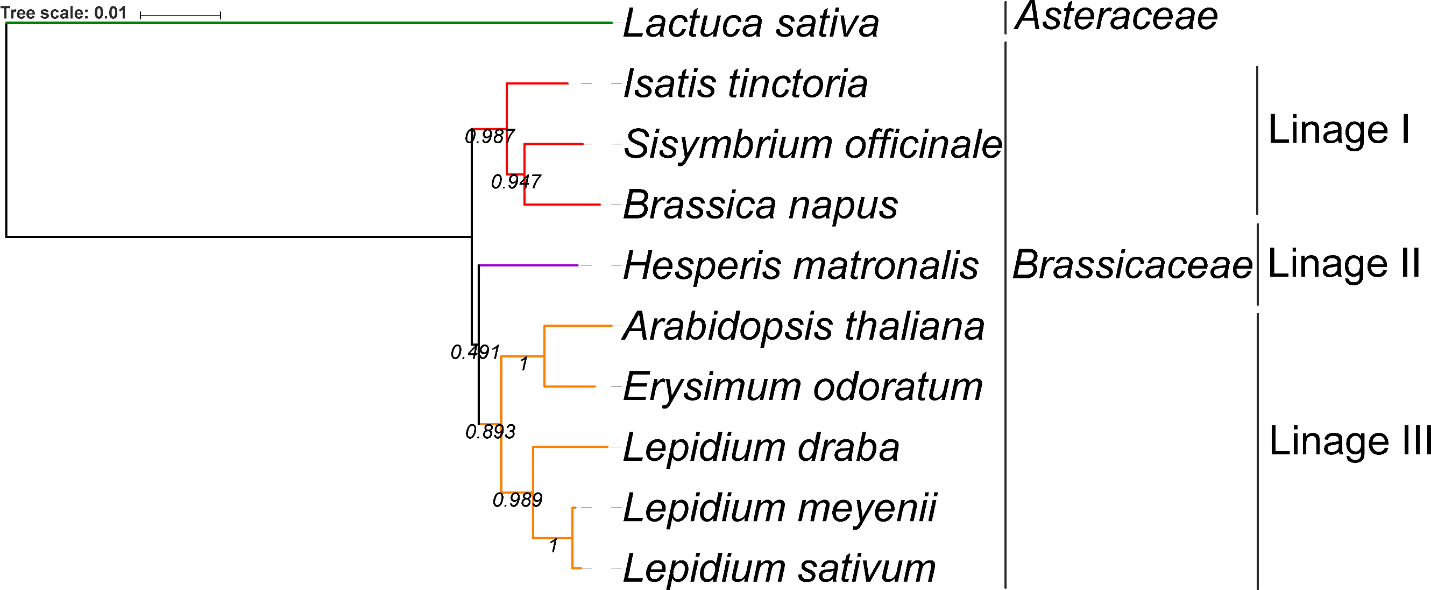


Fig. S4


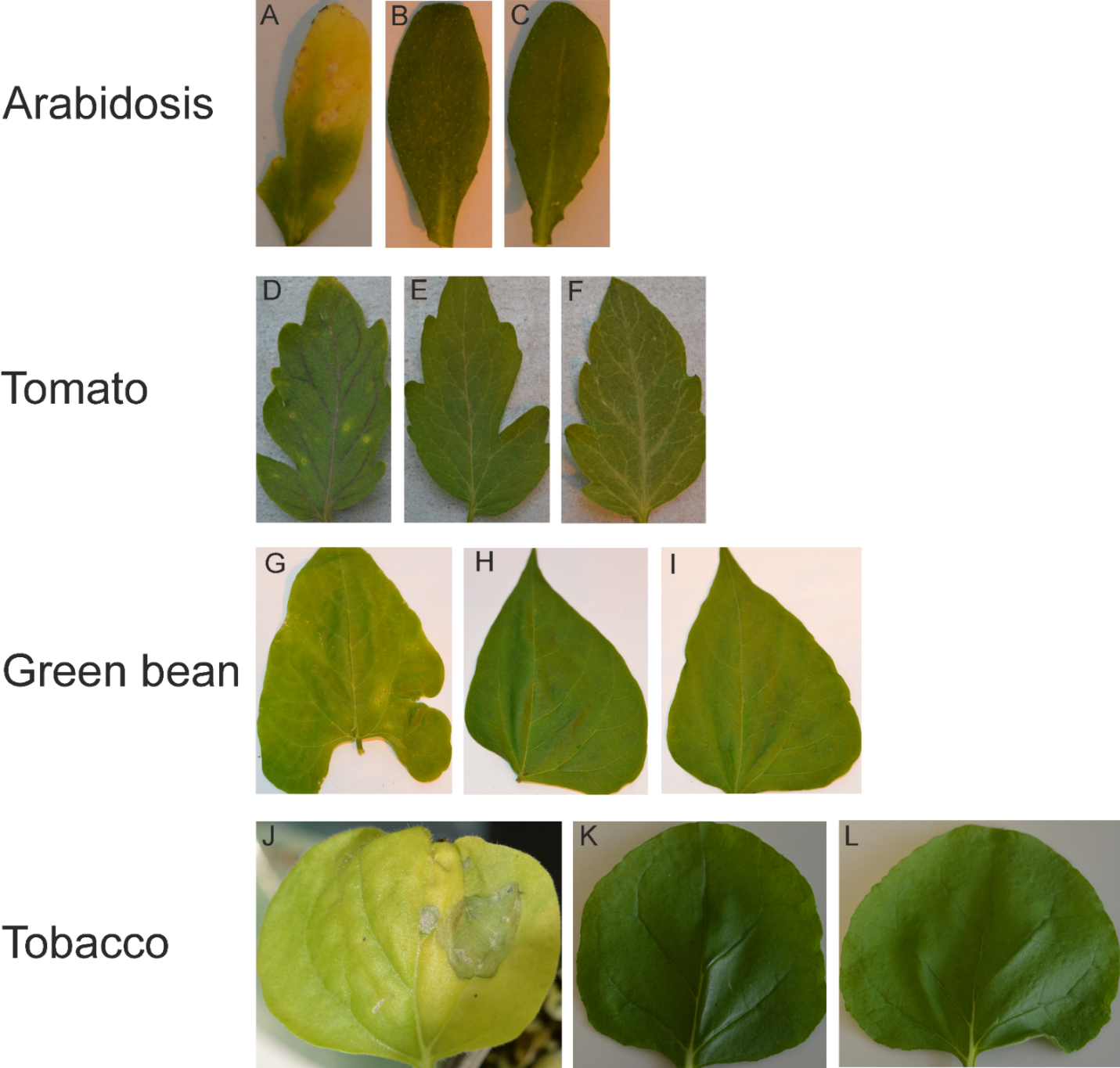


Fig. S5


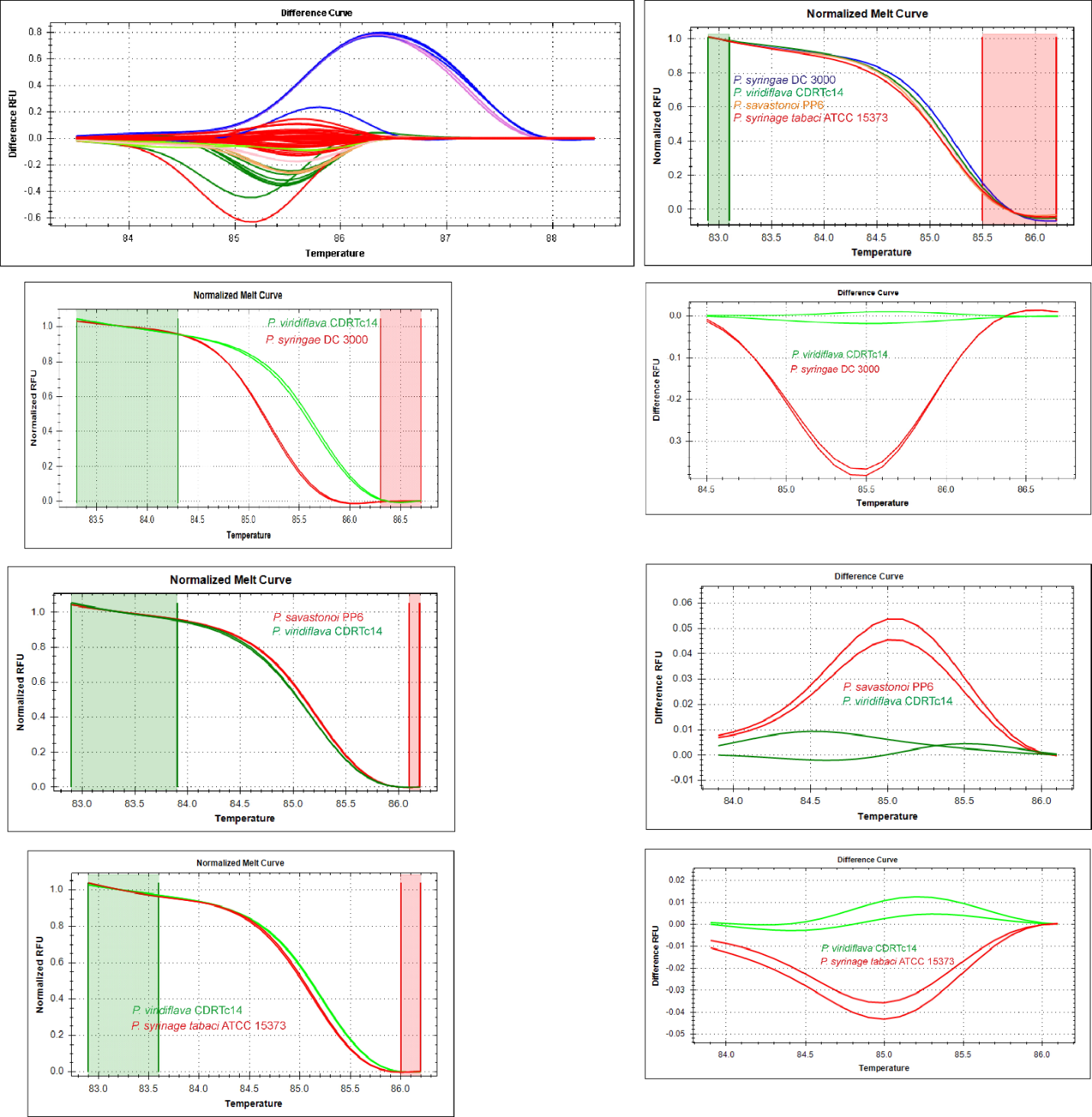


Fig. S6


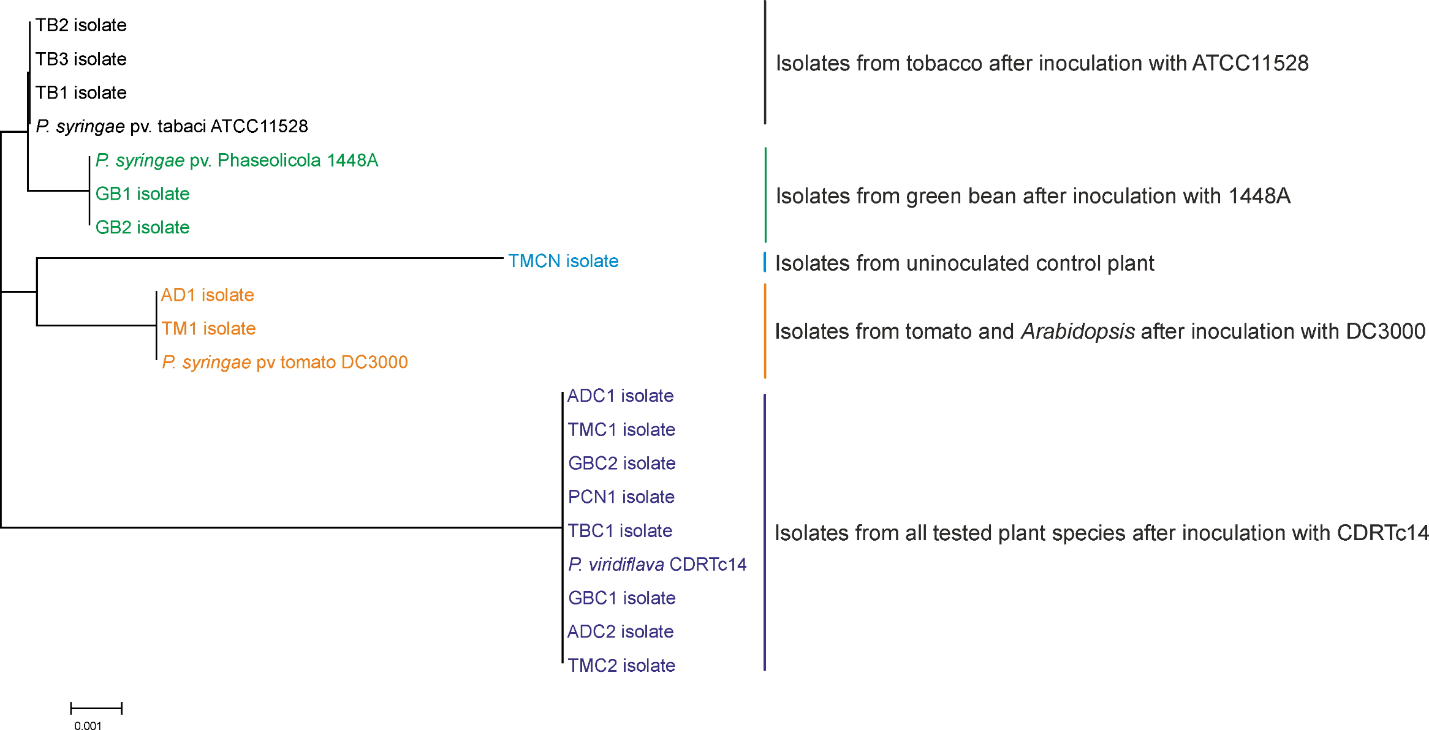


Fig. S7


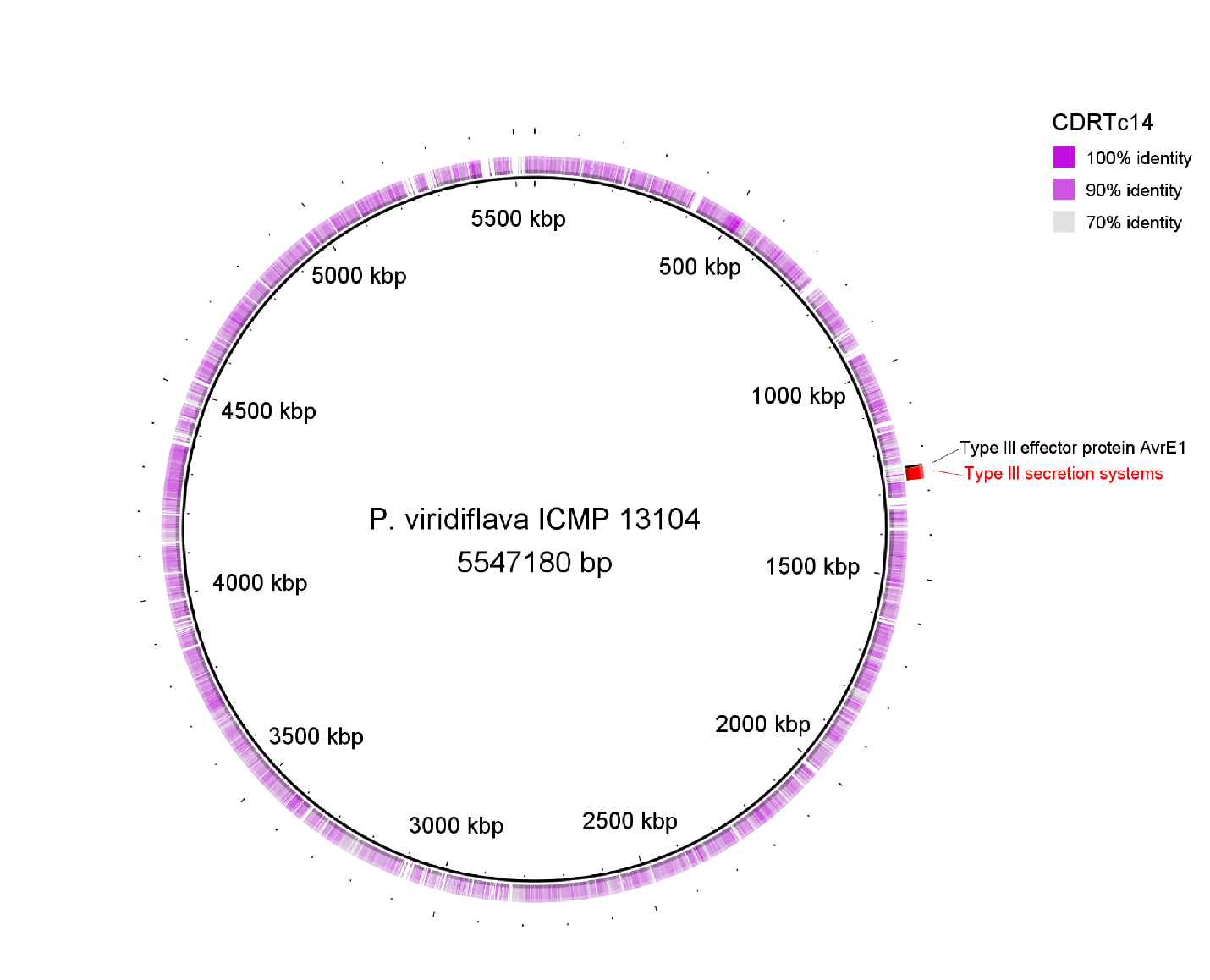


Fig. S8 (A)


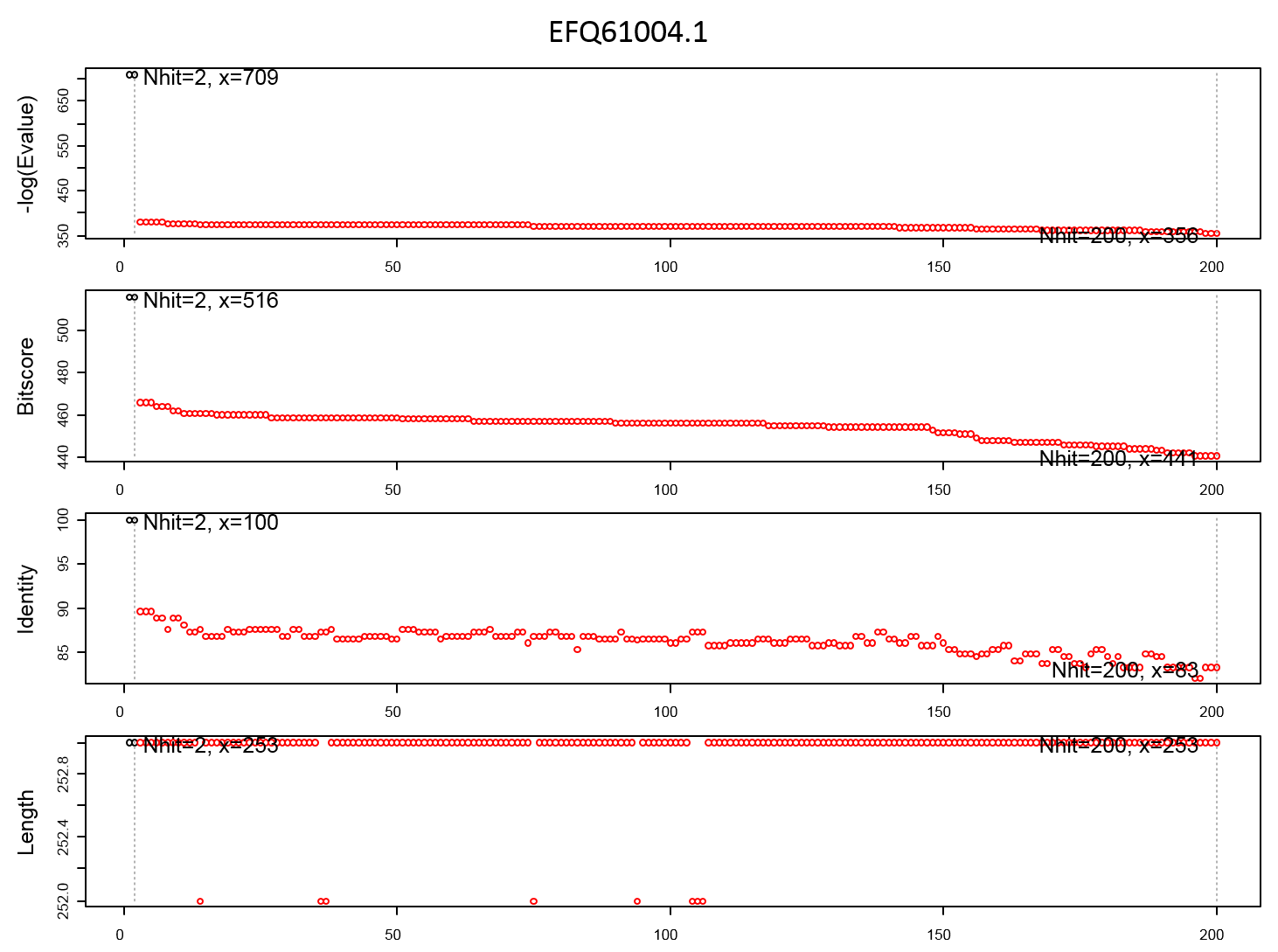


(B)


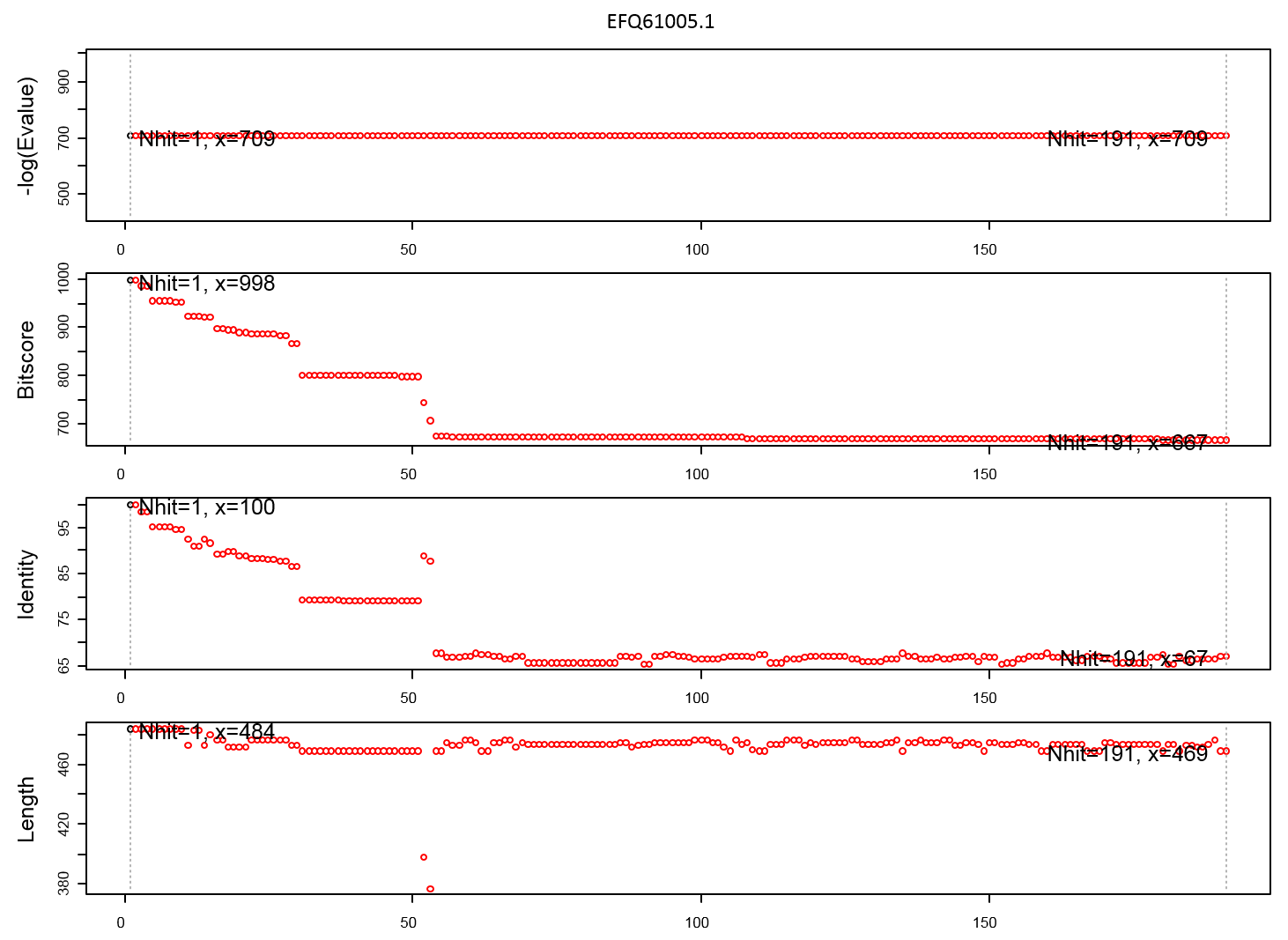


(C)


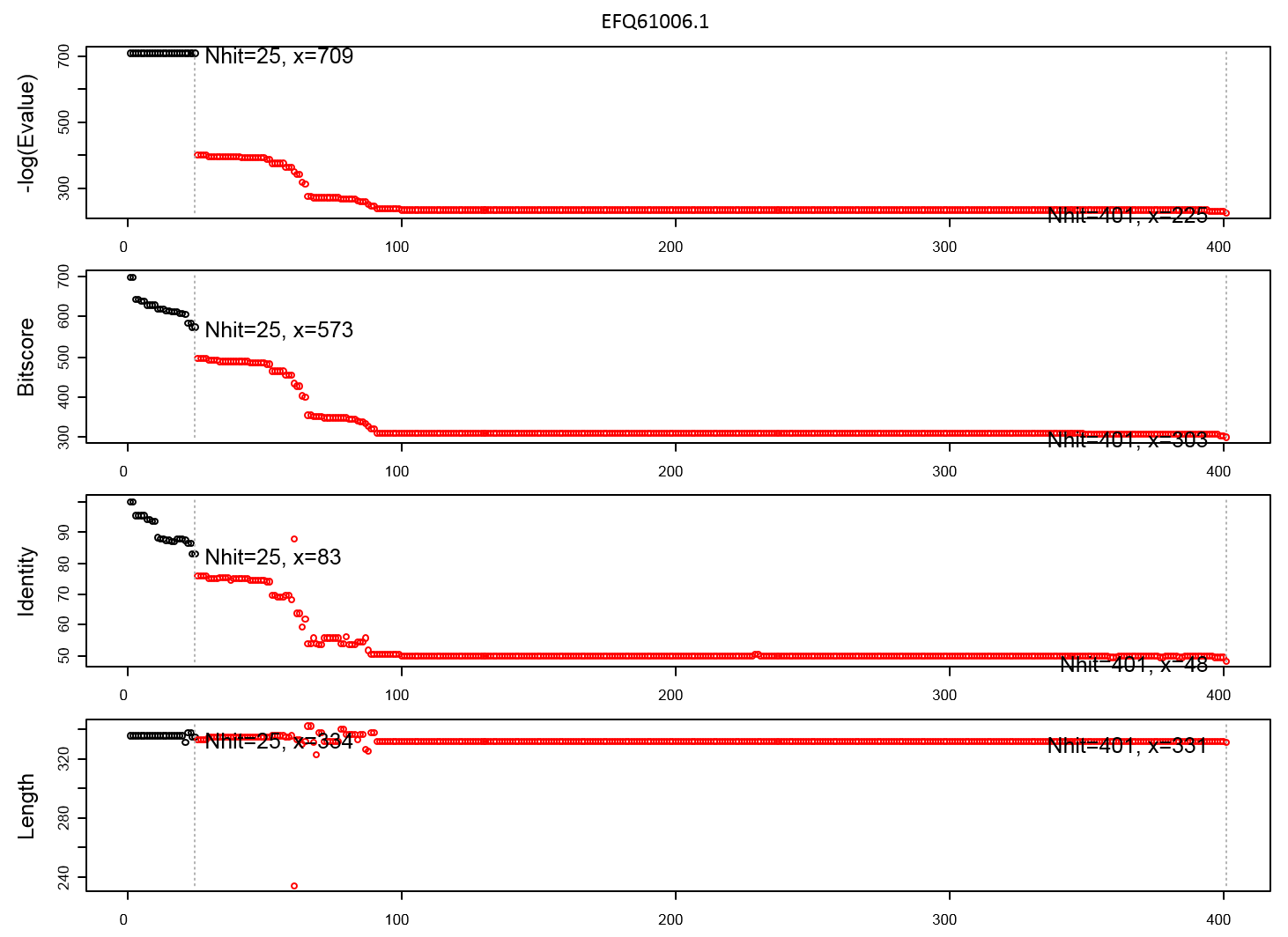


(D)


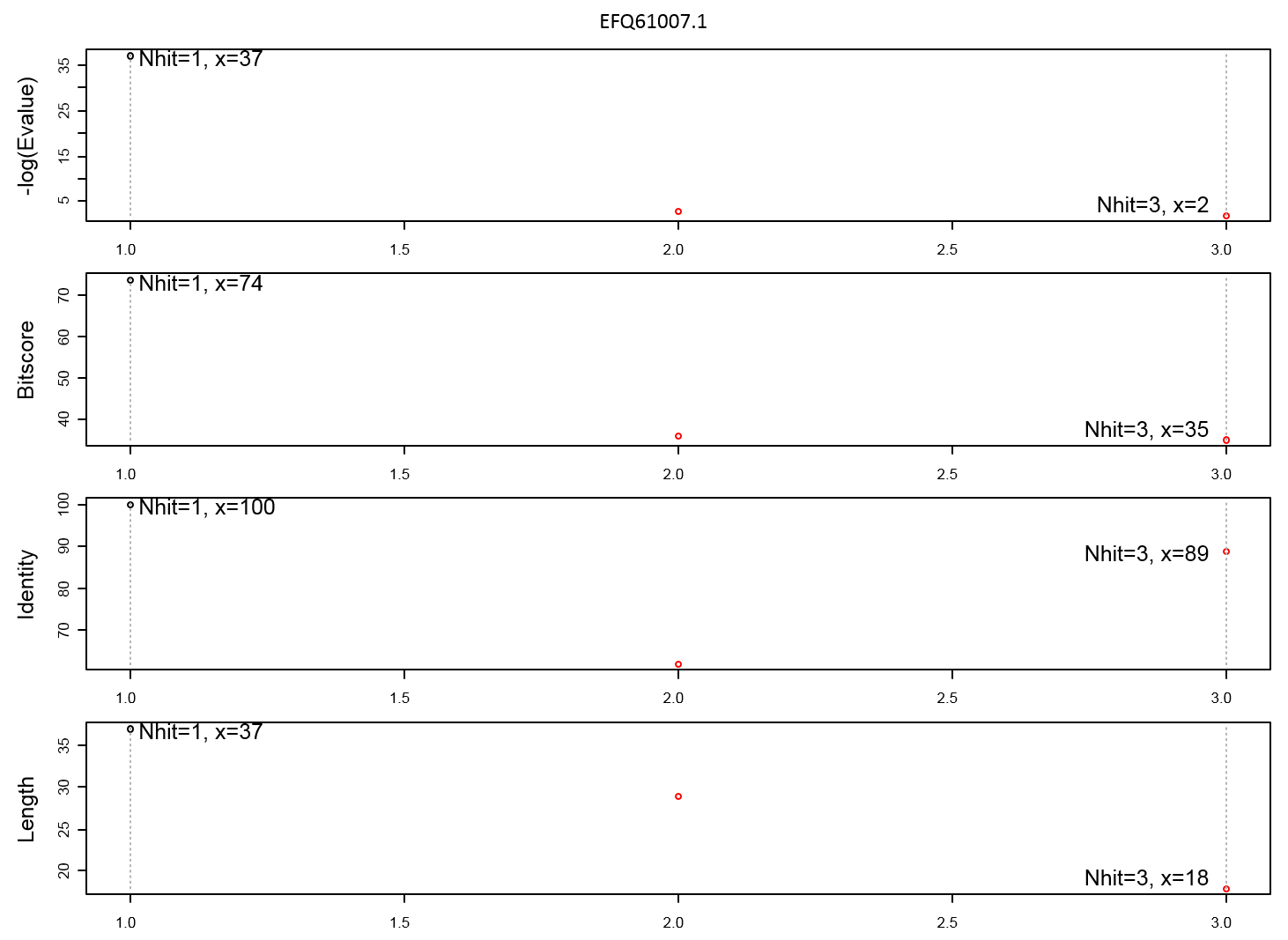


(E)


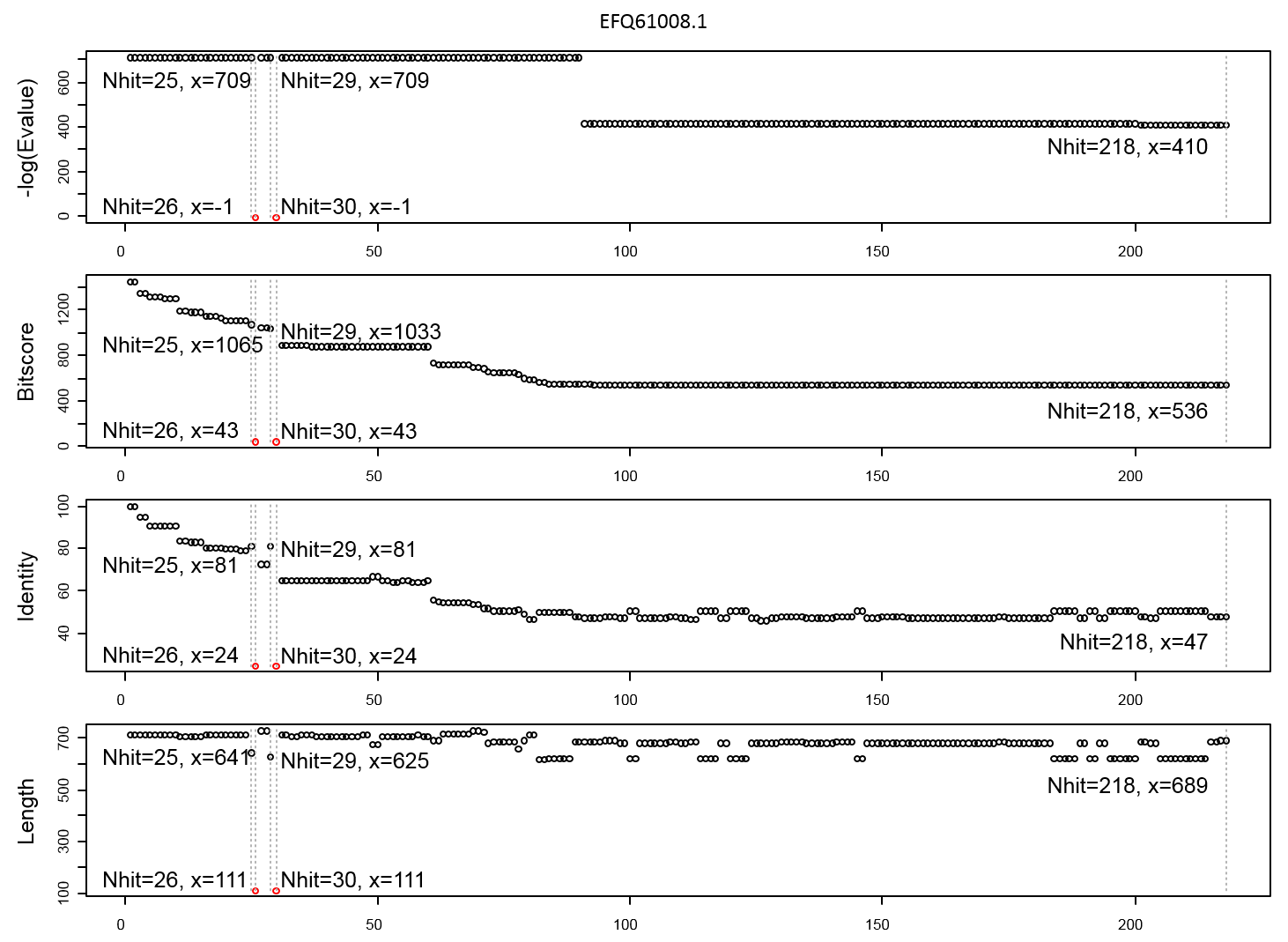


(F)


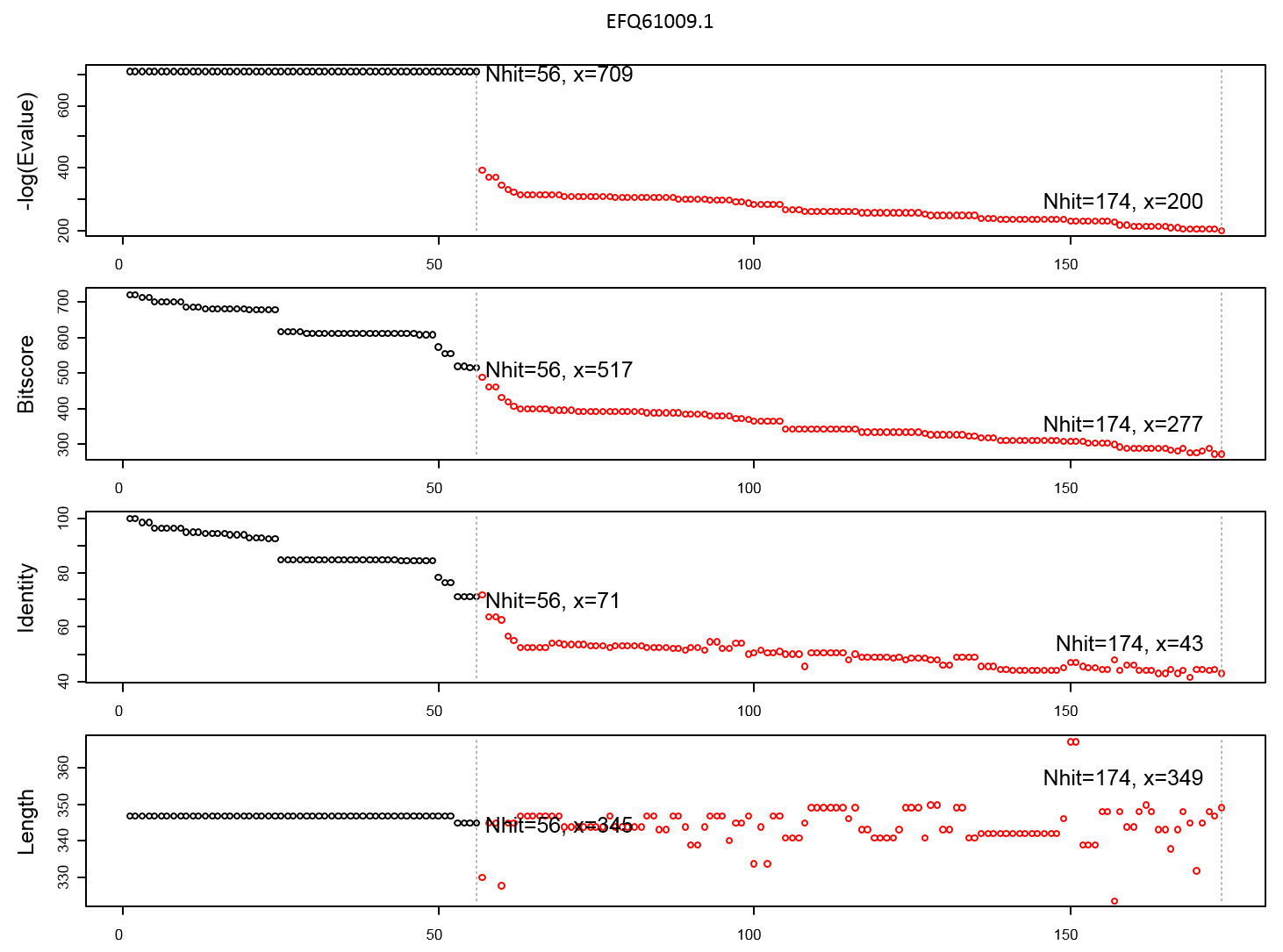


(G)


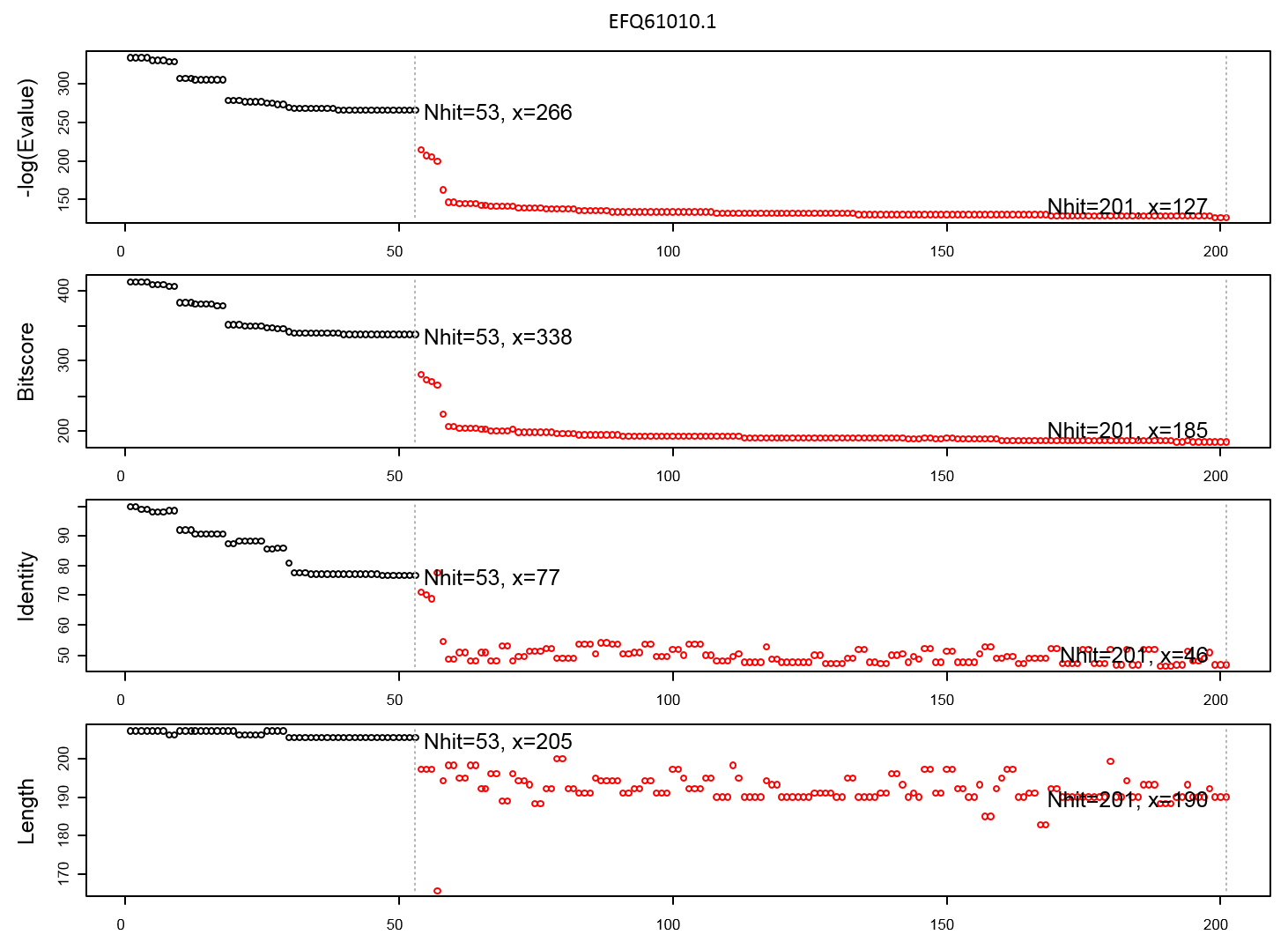


(H)


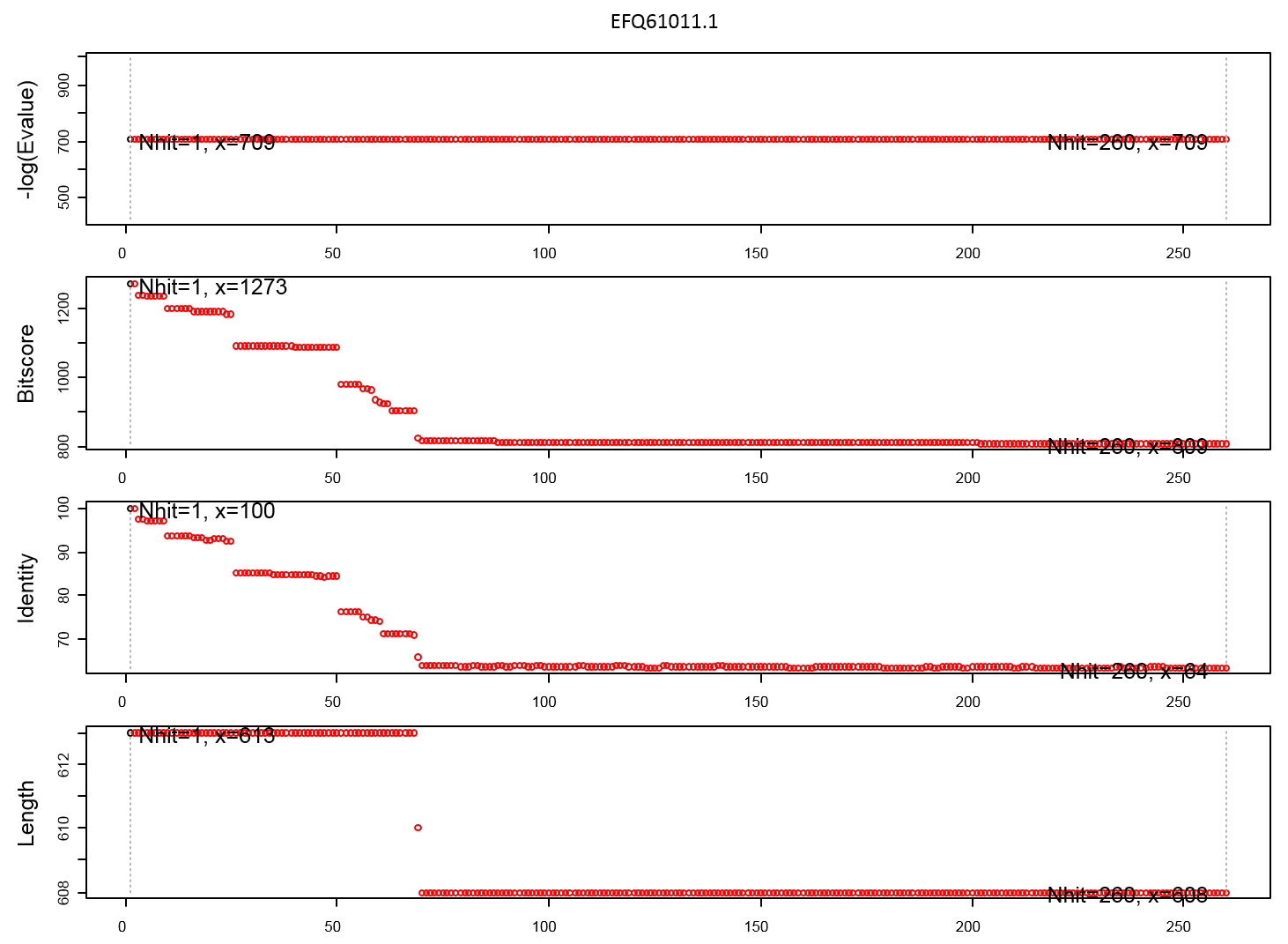


(I)


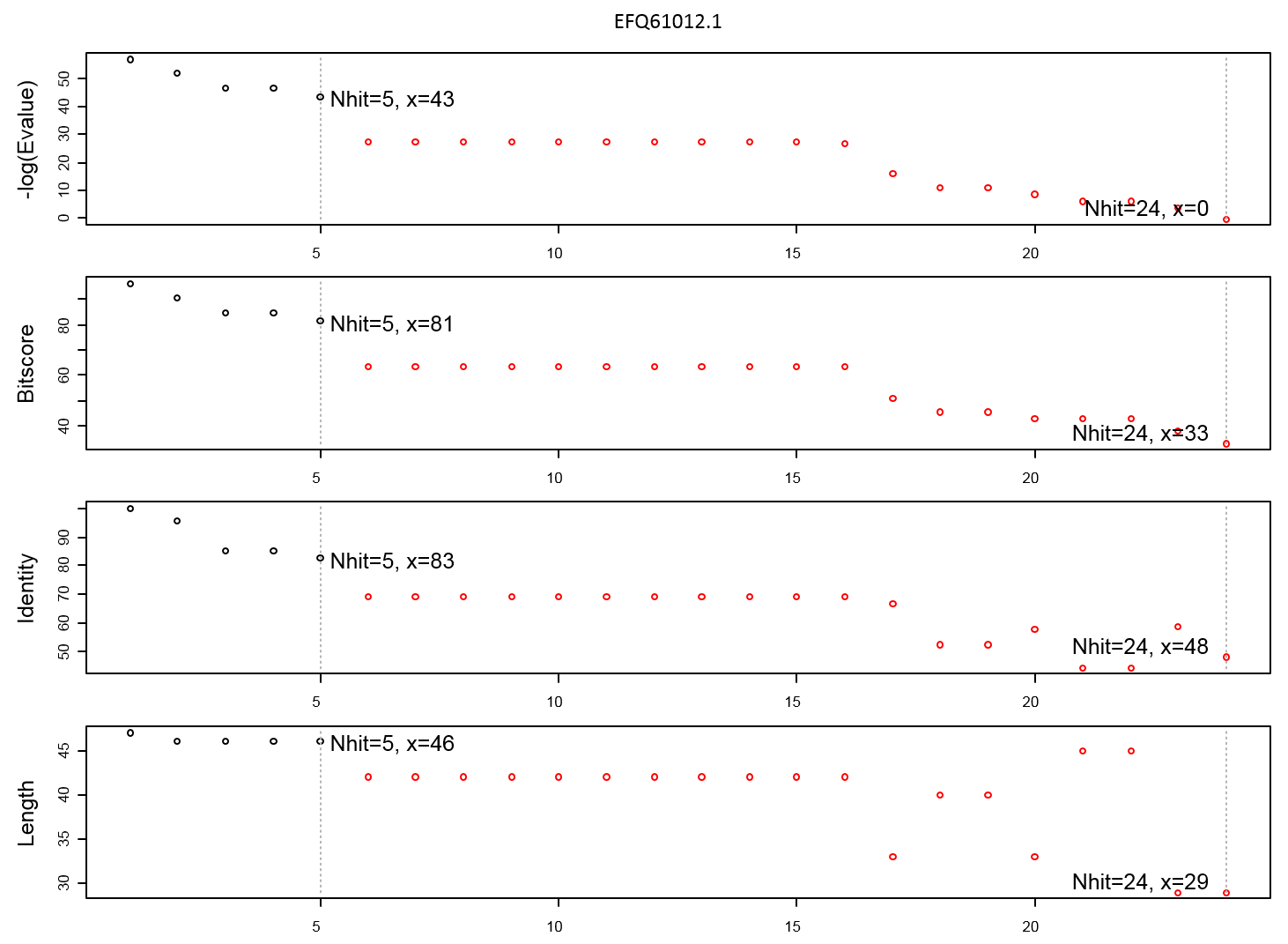


(J)


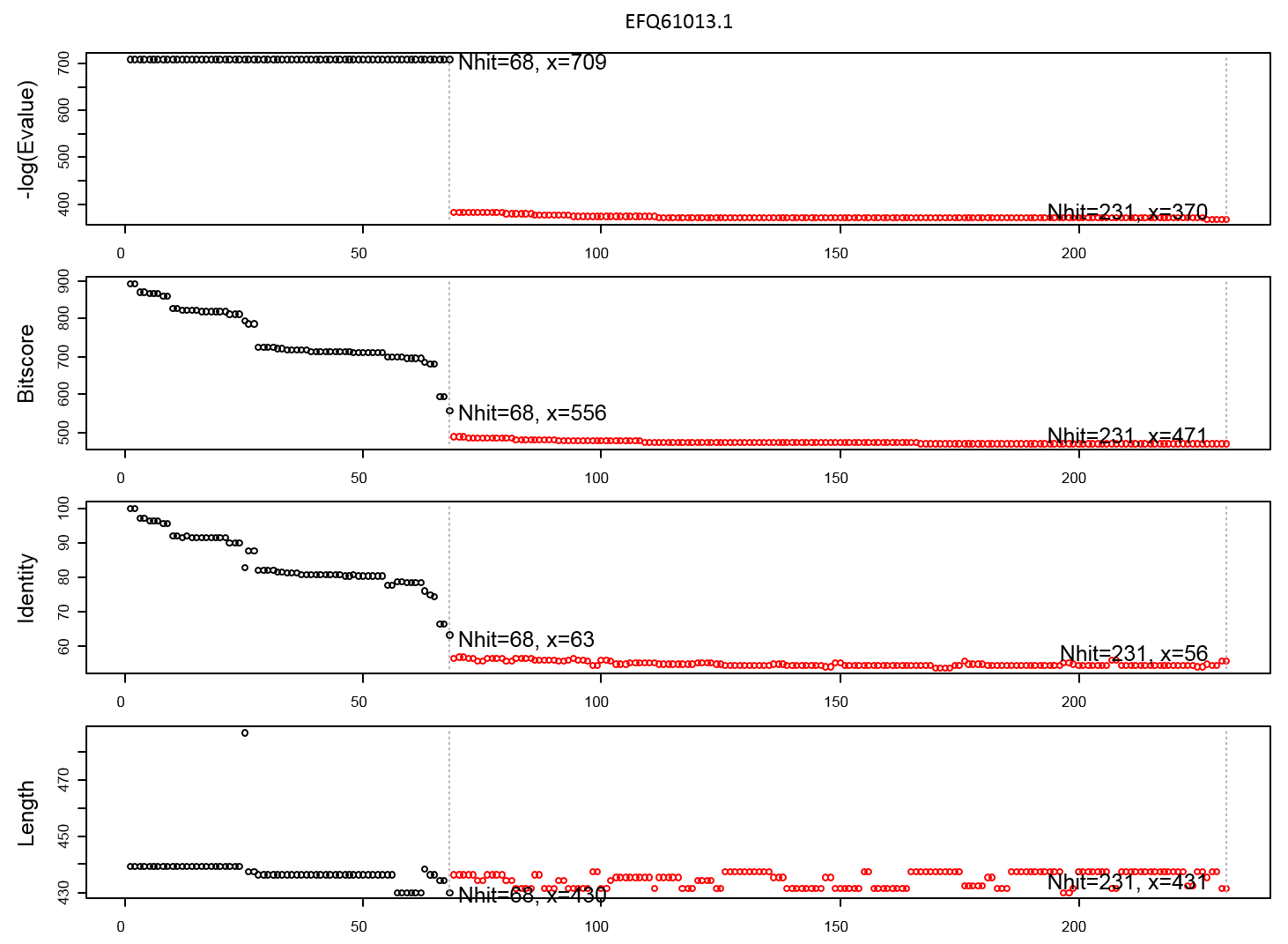


(K)


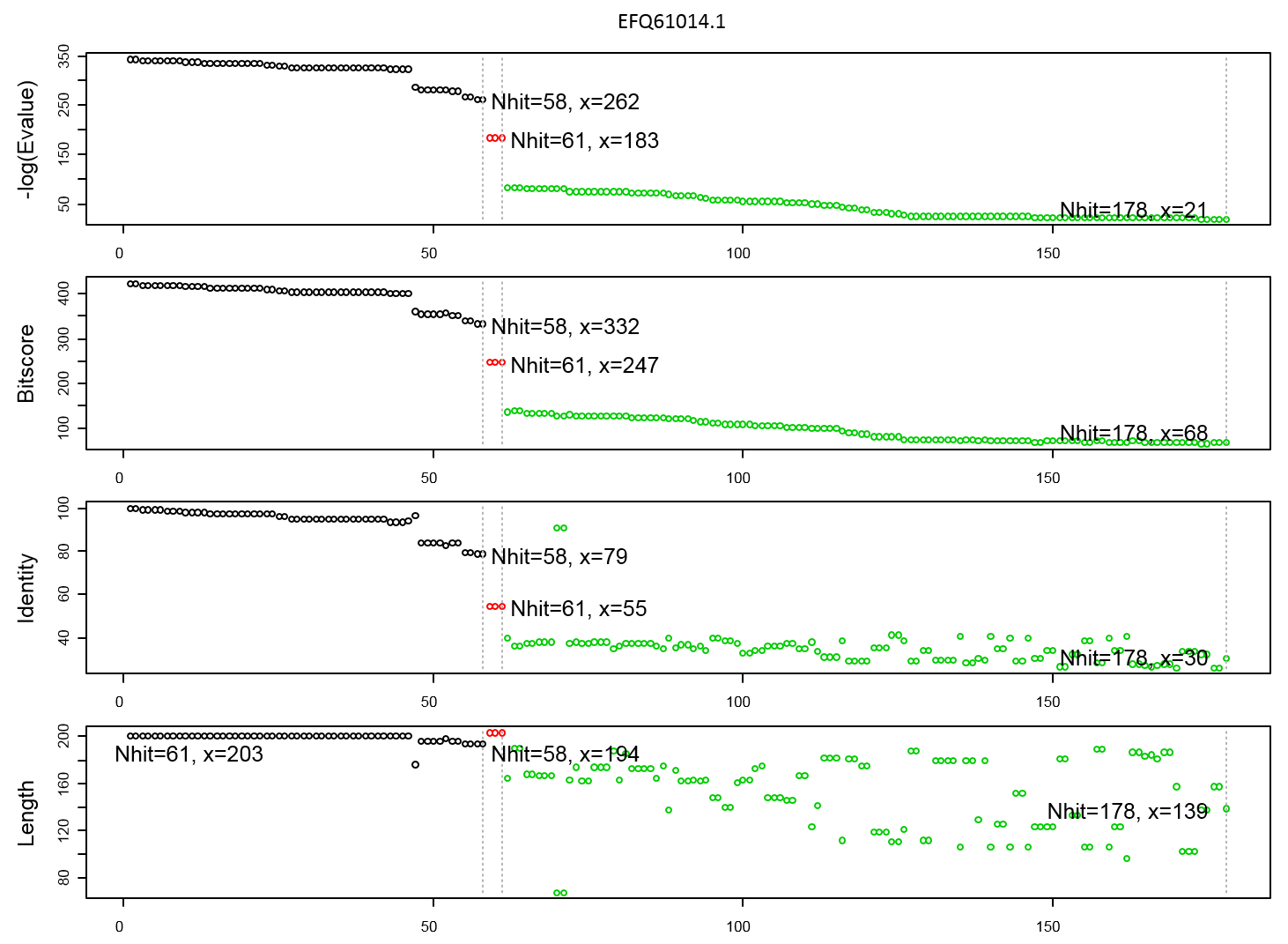


(L)


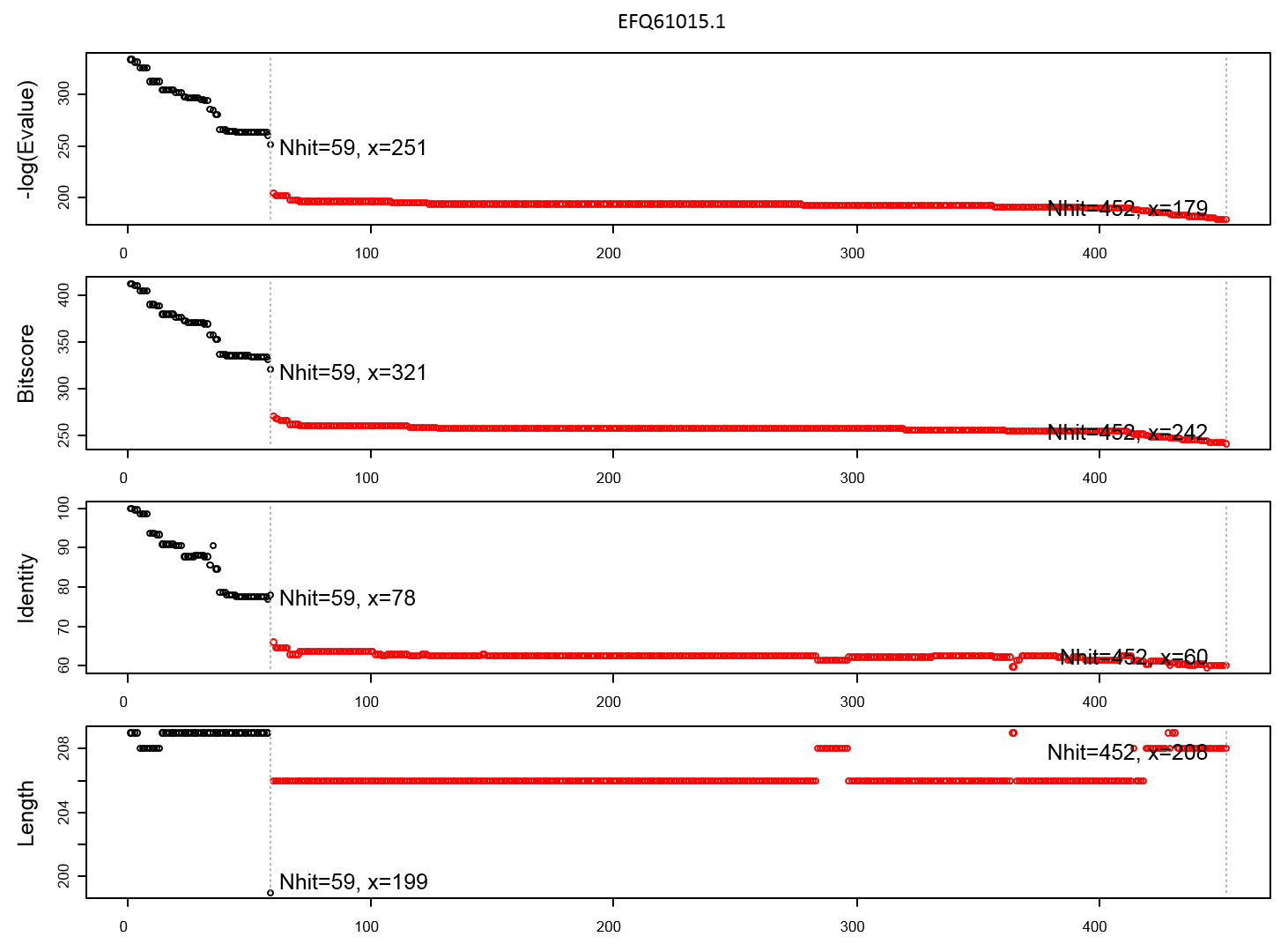


(M)


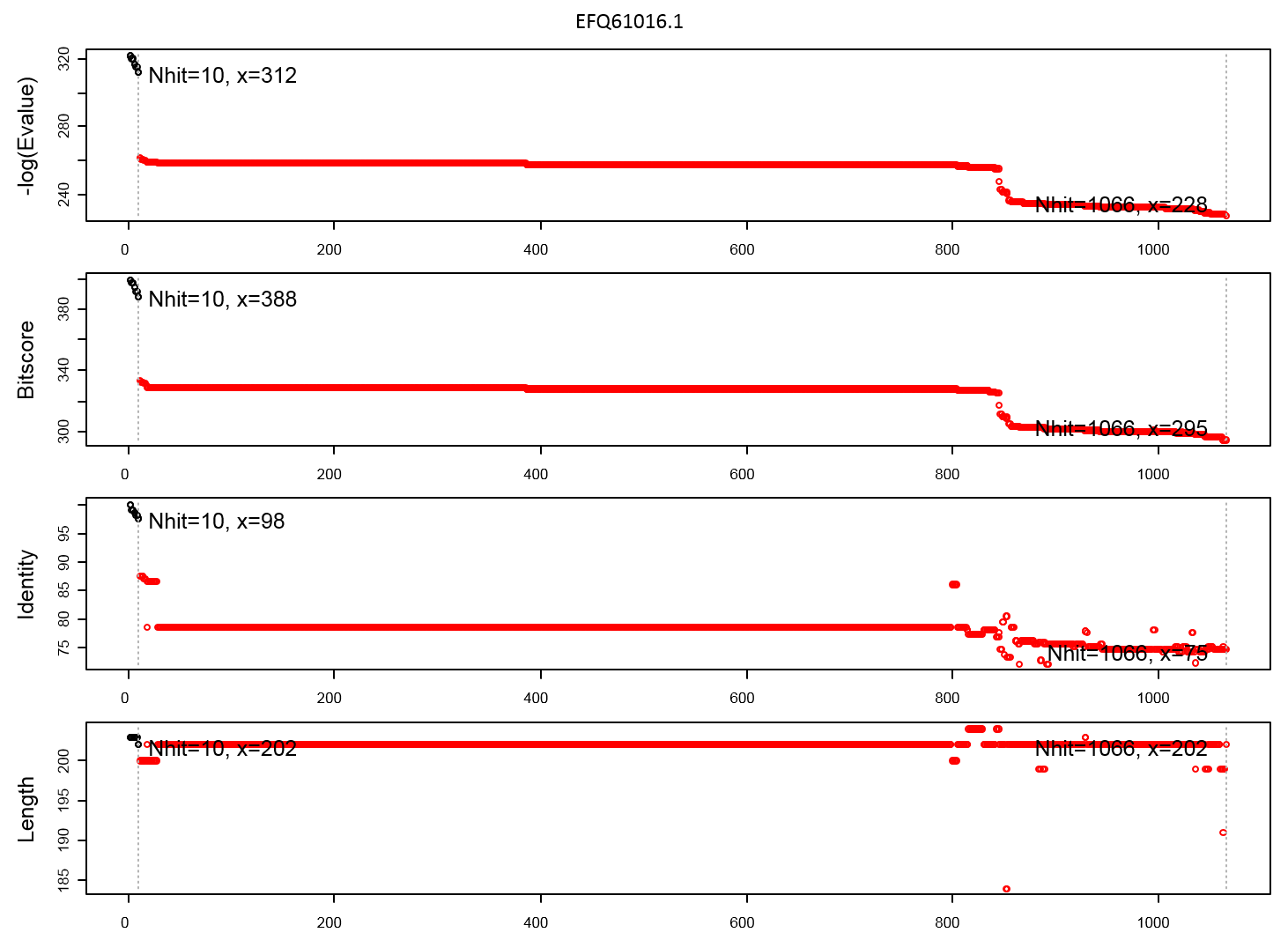


(N)


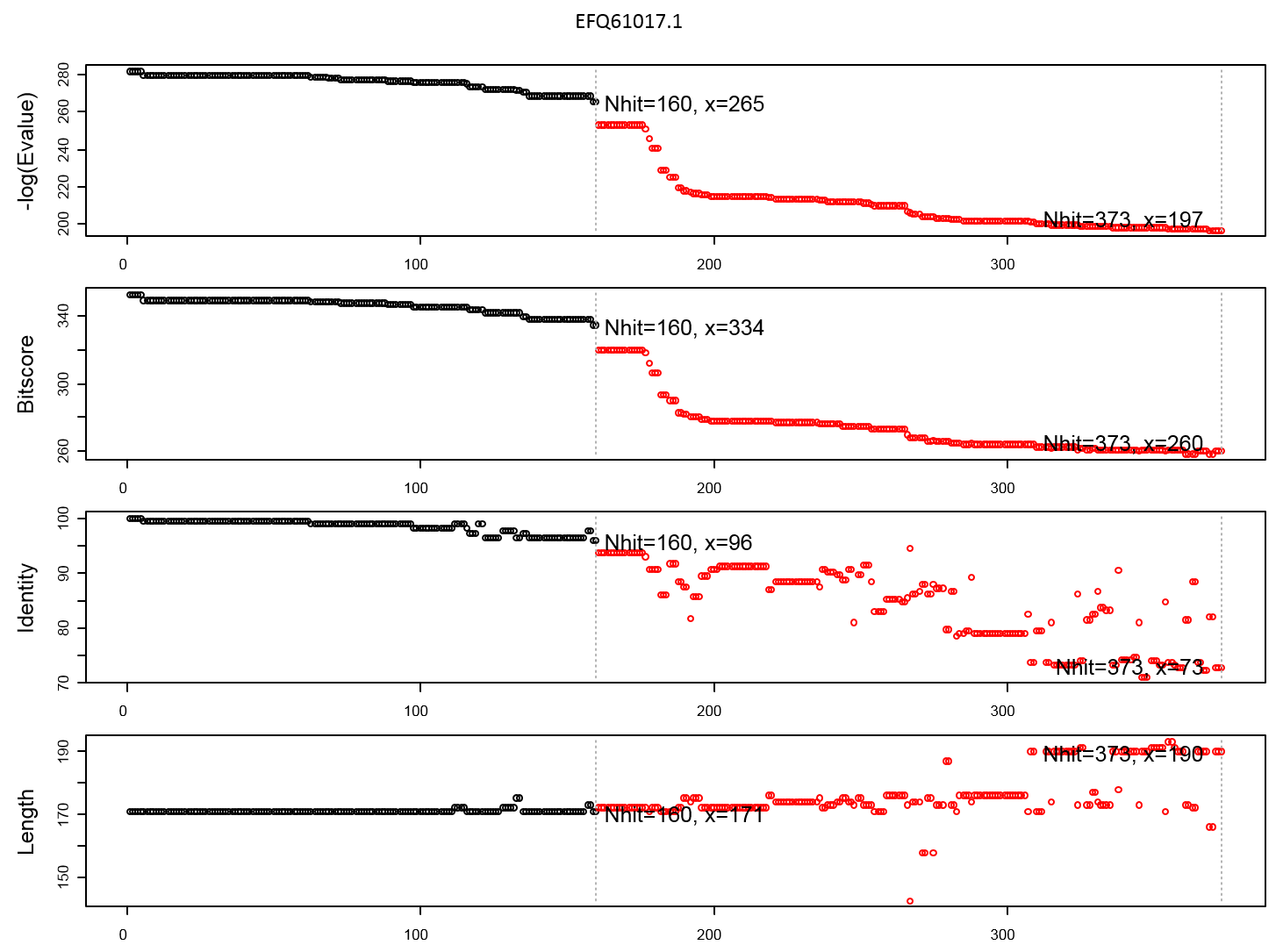


Fig. S9


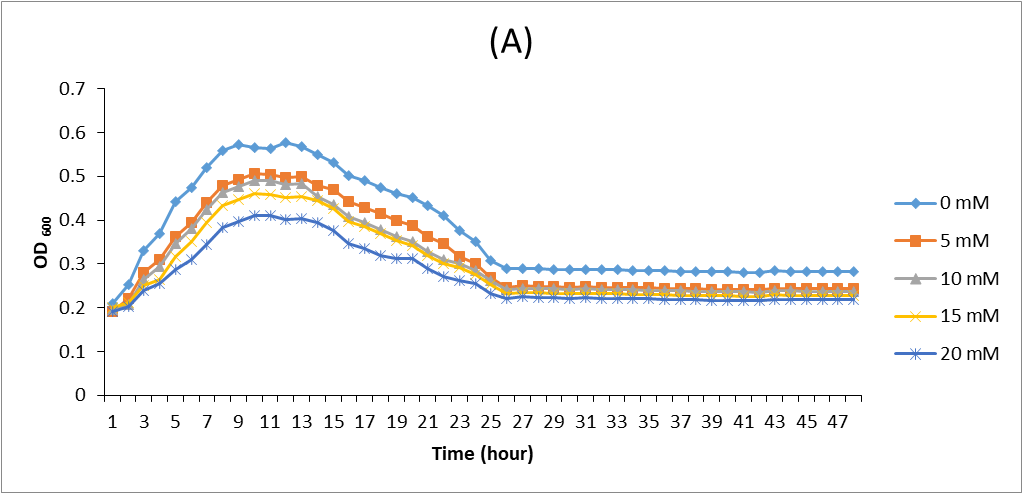


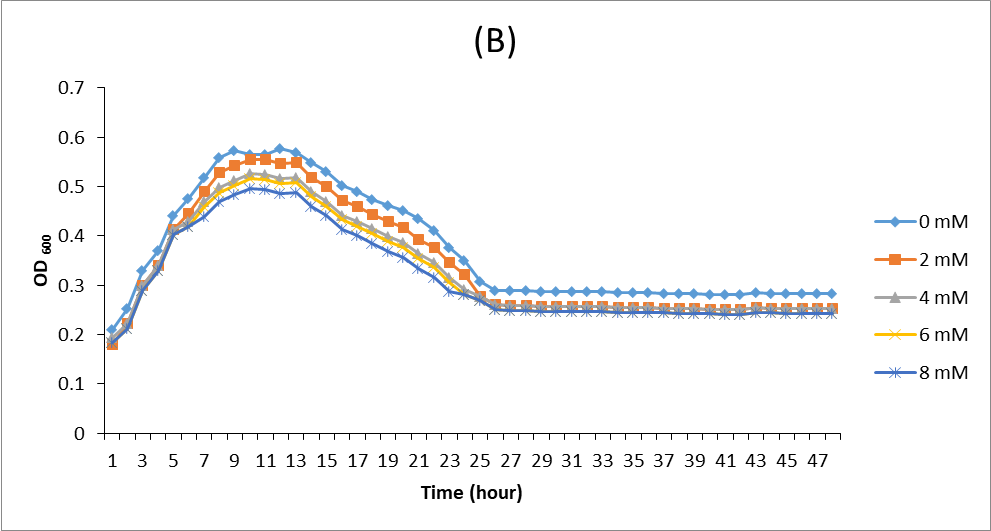


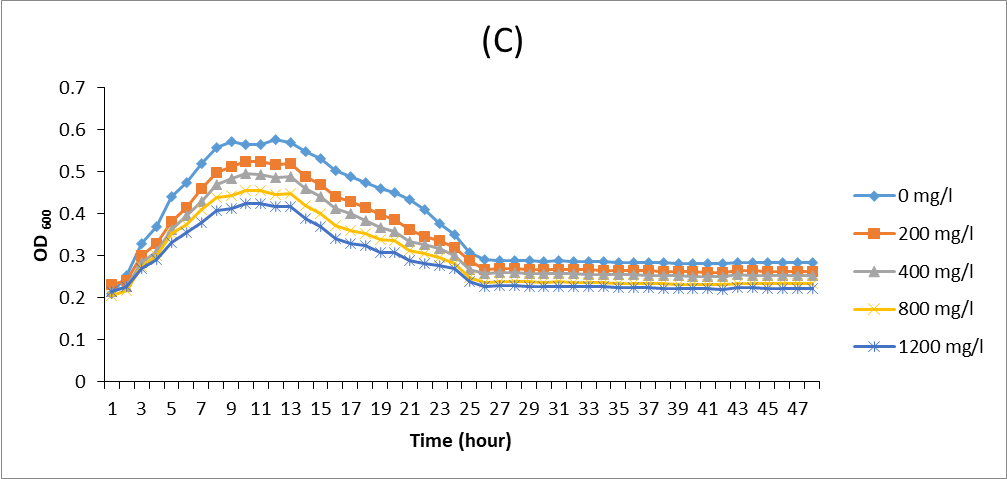

Supplement: Supplementary file 1 — Supplementary Material and Methods and Figures [file 41598_2017_16495_MOESM1_ESM.doc]
